# SMAD2/3 mediate oncogenic effects of TGF-β in the absence of SMAD4

**DOI:** 10.1038/s42003-022-03994-6

**Published:** 2022-10-07

**Authors:** Adrien Bertrand-Chapel, Cassandre Caligaris, Tanguy Fenouil, Clara Savary, Sophie Aires, Sylvie Martel, Paul Huchedé, Christelle Chassot, Véronique Chauvet, Victoire Cardot-Ruffino, Anne-Pierre Morel, Fabien Subtil, Kayvan Mohkam, Jean-Yves Mabrut, Laurie Tonon, Alain Viari, Philippe Cassier, Valérie Hervieu, Marie Castets, Alain Mauviel, Stéphanie Sentis, Laurent Bartholin

**Affiliations:** 1 TGF-β & Pancreatic Cancer Lab, Centre de Recherche en Cancérologie de Lyon (CRCL), Centre Léon Bérard, INSERM 1052, CNRS 5286, Université de Lyon, Université Claude Bernard Lyon 1, Lyon, France; 2 Hospices Civils de Lyon, Institute of Pathology, Groupement Hospitalier Est, Bron, France; 3 Ribosome, Translation and Cancer Lab, Centre de Recherche en Cancérologie de Lyon (CRCL), Centre Léon Bérard, INSERM 1052, CNRS 5286, Université de Lyon, Université Claude Bernard Lyon 1, Lyon, France; 4 Cell Death and Childhood Cancers Lab, Centre de Recherche en Cancérologie de Lyon (CRCL), Centre Léon Bérard, INSERM 1052, CNRS 5286, Université de Lyon, Université Claude Bernard Lyon 1, Labex DevWeCan, Institut Convergence Plascan, Lyon, France; 5 EMT and Cancer Cell Plasticity Lab, Centre de Recherche en Cancérologie de Lyon (CRCL), Centre Léon Bérard, INSERM 1052, CNRS 5286, Université de Lyon, Université Claude Bernard Lyon 1, Lyon, France; 6 Service de Biostatistiques, Hospices Civils de Lyon, Lyon France, Université de Lyon, Université Lyon 1, CNRS, Laboratoire de Biométrie et Biologie Évolutive, UMR 5558 Villeurbanne, France; 7 Hospices Civils de Lyon, Croix-Rousse University Hospital, Claude Bernard Lyon 1 University, Department of General Surgery & Liver Transplantation, Lyon, France; 8 Plateforme de bioinformatique Gilles Thomas, Fondation Lyon Synergie Cancer, Centre Léon Bérard, Lyon, France; 9 Département d’oncologie Médicale, unité de phase 1, Centre Léon Bérard, Lyon, France; 10 Team “TGF-ß and Oncogenesis”, Institut Curie, PSL Research University, INSERM 1021, CNRS 3347, Equipe Labellisée Ligue 2016, 91400 Orsay, France

**Keywords:** Cancer, Growth factor signalling

## Abstract

TGF-β signaling is involved in pancreatic ductal adenocarcinoma (PDAC) tumorigenesis, representing one of the four major pathways genetically altered in 100% of PDAC cases. TGF-β exerts complex and pleiotropic effects in cancers, notably *via* the activation of SMAD pathways, predominantly SMAD2/3/4. Though SMAD2 and 3 are rarely mutated in cancers, SMAD4 is lost in about 50% of PDAC, and the role of SMAD2/3 in a SMAD4-null context remains understudied. We herein provide evidence of a SMAD2/3 oncogenic effect in response to TGF-β1 in SMAD4-null human PDAC cancer cells. We report that inactivation of SMAD2/3 in SMAD4-negative PDAC cells compromises TGF-β-driven collective migration mediated by FAK and Rho/Rac signaling. Moreover, RNA-sequencing analyses highlight a TGF-β gene signature related to aggressiveness mediated by SMAD2/3 in the absence of SMAD4. Using a PDAC patient cohort, we reveal that SMAD4-negative tumors with high levels of phospho-SMAD2 are more aggressive and have a poorer prognosis. Thus, loss of SMAD4 tumor suppressive activity in PDAC leads to an oncogenic gain-of-function of SMAD2/3, and to the onset of associated deleterious effects.

## Abstract (web summary)

In pancreatic ductal adenocarcinoma cells and patient tissue, SMAD2/3 is shown to mediate oncogenic effects of TGF-β in the absence of SMAD4.

## Introduction

TGF-β1 (further referred to as TGF-β) is a secreted pleiotropic cytokine involved in several physiological and pathological processes. The “canonical” TGF-β signaling pathway is mediated by SMAD transcription factors. Upon binding of TGF-β to its serine/threonine kinase receptors (TβR I and II), R-SMADs, i.e., SMAD2 and SMAD3, are phosphorylated and interact with the co-SMAD (common SMAD), namely SMAD4. The SMAD2/3/4 complex accumulates inside the nucleus and binds to the promoter of target genes to regulate their transcription^1^. TGF-β also activates “non-canonical” pathways independently of SMAD proteins such as mitogen-activated protein kinases (MAP kinases)^2^, phosphatidylinositol 3-kinases (PI3K)/AKT^3^, small G-proteins such as Rac and Rho, and reactive oxygen species (ROS)^4^.

TGF-β was shown to have both tumor suppressive properties, in the early stages of transformation, and a tumor promoting role in more advanced stages of the disease^5–7^. Tumor suppressive effects are classically associated with the activation of the “canonical” pathway in cancer cells by inducing the transcription of genes involved in cytostasis (p21, p15) and apoptosis (Bim), and by inhibiting the transcription of oncogenes (c-myc)^8^. However, the “canonical” pathway is also involved in TGF-β-mediated epithelial-mesenchymal transition (EMT), invasiveness and proliferation of cancer cells in vitro^7,9,10^ and in vivo^11^. TGF-β exerts further oncogenic effects via the induction of “non-canonical pathways”, such as the ERK or PI3K-Akt pathways. The oncogenic effects of TGF-β notably result in the activation of genes involved in EMT^12,13^, or in matrix remodeling^14^. In addition, TGF-β also affects stromal cells leading to immunosuppression, increased angiogenesis and activation of cancer-associated fibroblasts (CAFs)^5,15–17^. Moreover, as an illustration of a higher level of complexity, the crosstalk between SMAD and non-SMAD signaling is particularly important in the control of cell migration and invasion^16^.

In the context of pancreatic ductal adenocarcinoma (PDAC), the TGF-β pathway is one of only four signaling pathways that is genetically altered (with at least one mutation) in 100% of PDAC^18^. TGF-β ligands are commonly overexpressed in PDAC, and the hyperactivation of TGF-β signaling is correlated with poor prognosis^19,20^. Interestingly, SMAD2/3 inactivating mutations are scarce in cancers, notably in PDAC^15,21,22^. Conversely, SMAD4 deficiency is observed in ~50% of PDAC and is associated with a poor overall survival^10,23^. These observations suggest that in the absence of SMAD4, SMAD2/3 may have yet uncharacterized deleterious effects, yet uncharacterized. In response to TGF-β, SMAD2 and SMAD3 can accumulate inside the nucleus in the absence of SMAD4, suggesting that they may regulate specific SMAD4-independent transcriptional programs^24–27^, possibly by interacting with nuclear factors such as IKK and TIF1γ^28,29^. Besides, a study by David et al. showed that R-SMAD induces Sox4 expression in a SMAD4-independent manner in SMAD4-null PDAC cells, and cooperates with Klf5, leading to tumor promotion^30^.

Taking these elements into account, we speculated that SMAD2 and SMAD3 may behave as oncogenic factors in a SMAD4-negative context. Since PDAC represents a valuable model to characterize SMAD2 and SMAD3 functions after SMAD4 inactivation during the natural course of the disease, we generated a canonical-deficient SMAD2/3 double knockout BxPC-3 cell line (also naturally devoid of SMAD4) to test this hypothesis. The TGF-β1 ligand was used throughout the study. Functional in vitro and in vivo experiments and RNA sequencing were performed to explore the aggressiveness of these cells, prior to correlating SMAD2 activation level and SMAD4 status with the clinical status of patient in a large PDAC cohort.

## Results

### Generation of double SMAD2/SMAD3-negative pancreatic cancer cell in a SMAD4-negative context

**Fig. 1:**
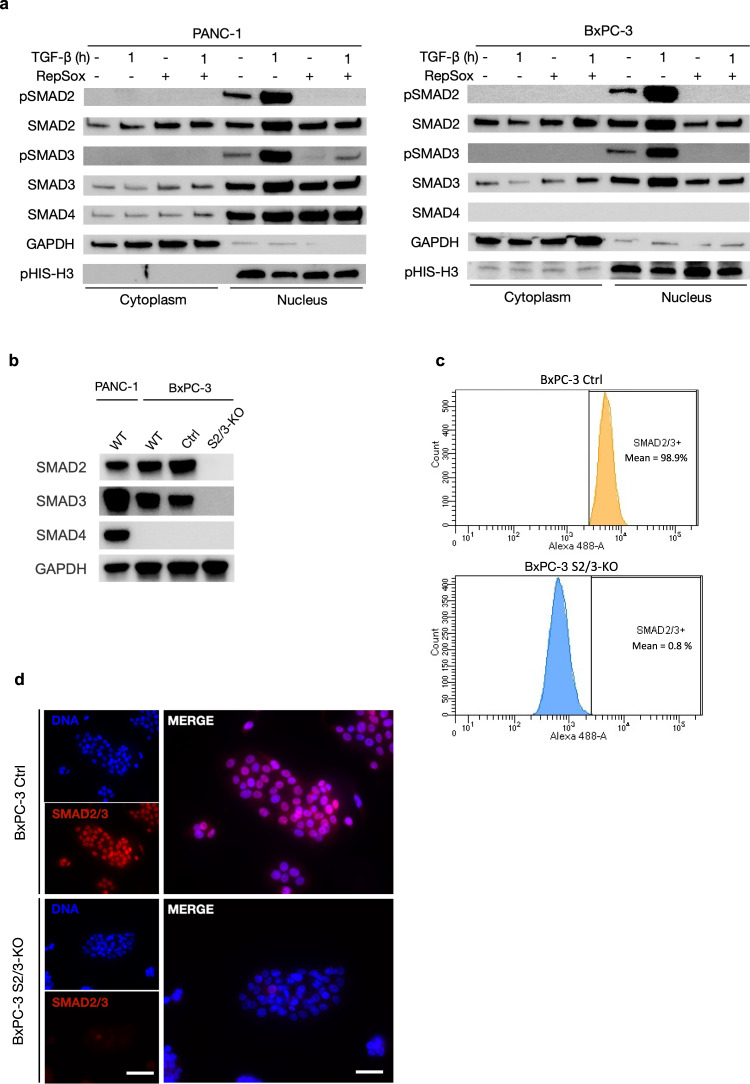
Generation of a double SMAD2/SMAD3 knockout BxPC-3 (SMAD4-negative) pancreatic cancer cell line. **a** Immunoblot of phospho-SMAD2 (pSMAD2), SMAD2, phospho-SMAD3 (pSMAD3), SMAD3 and SMAD4 after treatment with TGF-β and/or TGF-βR1 kinase activity inhibitor (RepSox) for 1 h on cytoplasmic (GAPDH as control) and nuclear fractions (phospho-histone H3 (pHIS-H3) as control) prepared from SMAD4-positive PANC-1 cells (left panel) and SMAD4-negative BxPC-3 cells (right panel). One representative image out of 3 independent experiments is shown. **b** Immunoblot of SMAD2, SMAD3, SMAD4 and GAPDH on PANC-1 (SMAD4^+^), BxPC-3 (SMAD4^-^) wild-type (WT), control (Ctrl) and SMAD2/SMAD3-knockout (S2/3-KO) cells obtained by CRISPR/Cas9 genome editing technique. One representative image out of 3 independent experiments is shown. **c** Flow cytometric analysis of SMAD2/3 performed on control and S2/3-KO BxPC-3 cells. SMAD2/3 *ratio* was determined as the mean value of three independent experiments. **d** Immunofluorescent micrographs of SMAD2/SMAD3 staining on control and S2/3-KO BxPC-3 cells treated with TGF-β (1 h). Nuclei were counterstained in blue with DAPI. Representative images out of 3 independent experiments are shown. Scale bar (left-hand images) = 50 µm. Scale bar (right-hand images) = 20 µm.

We initially sought to confirm previous findings on the effect of TGF-β on the sub-cellular localization of SMAD2/3 in SMAD4-positive (PANC-1) and SMAD4-negative (BxPC-3 and Capan-1) human PDAC cell lines^24,27^. Immunoblot analysis of the nuclear and cytoplasmic cell fractions demonstrated that in response to TGF-β (1 h or 24 h), phosphorylated SMAD2/3 (pSMAD2/pSMAD3) accumulate in the nucleus of both SMAD4-positive (PANC-1) and SMAD4-negative cells (BxPC-3 and Capan-1), this nuclear location being prevented by adding the selective inhibitor of TβRI kinase, RepSox (Figs. 1a and S1a, b). Hence, SMAD2/3 phosphorylation and their nuclear accumulation in the presence of TGF-β occurs despite the absence of SMAD4 and is directly dependent on TβRI kinase activity.

We decided to investigate SMAD2/3 TGF-β-induced roles in the absence of SMAD4 in the BxPC-3 cell line, which is naturally devoid of SMAD4. To do so, we have generated double knock out (KO) for SMAD2 and SMAD3 by CRISPR/Cas9 gene editing, as well as single SMAD2 or 3 KO served as controls. These cell lines will hereafter be referred to as S2/3-KO, S2-KO, or S3-KO BxPC-3 cells. Loss of SMAD2/3 expression was validated by immunoblot analysis (Figs. 1b and S1c) and by flow-cytometry (Fig. 1c). FACS analysis detected 98.9% of SMAD2/3-positive cells in control BxPC-3 and 0.8% of SMAD2/3-positive cells in S2/3-KO BxPC-3 cells. Immunofluorescence (IF) experiments confirmed both the capacity of TGF-β to induce SMAD2/3 nuclear accumulation in WT BxPC-3 cells, and the absence of both cytoplasmic and nuclear SMAD2/3 in S2/3-KO BxPC-3 cells treated with TGF-β (Fig. 1d). Sequencing unveiled a specific deletion of both SMAD2 and SMAD3 in S2/3-KO BxPC-3 cells.

Importantly, TGF-β-receptors I/II levels were not affected by SMAD2/3 KO (Fig. S1d), ruling out a possible downregulation of the receptors as a basis for altered signaling upon SMAD2/3 KO. Consistently, activation of non-canonical TGF-β pathways was slightly delayed but was not significantly affected after 1 h and 24 h incubation with TGF-β in S2/3-KO BxPC-3 cells (Fig. S1e). TGF-β was shown to activate not only SMAD2/3-mediated signaling, but also, to a lesser extent, SMAD1/5-mediated BMP (bone morphogenetic proteins) signaling^31–33^. Here however, SMAD1/5/9 was similarly phosphorylated in response to TGF-β, in control BxPC-3 and S2/3-KO BxPC-3 cells (Fig. S1f). No significant differences were observed in terms of proliferation and apoptosis after TGF-β treatment between control and S2/3-KO BxPC-3 cells (Fig. S1g, h).

To strengthen our characterization of the impact of SMAD2/3 in a SMAD4-negative context, we also used a RNA interference (shRNA) strategy in SMAD4-negative pancreatic BxPC-3 and Capan-1 cells (Fig. S1i, j), thereafter referred to as S2-KD, S3-KD, or S2/3-KD cells.

Thus, using both CRISPR/Cas9 gene editing and RNA interference strategies, we created genuine pancreatic cell line models to study the impact of double inactivation of SMAD2 and SMAD3 in a SMAD4-negative context.

### Double invalidation of SMAD2/3 impairs pancreatic cell migration and invasion potential in response to TGF-β in a SMAD4-negative context

**Fig. 2:**
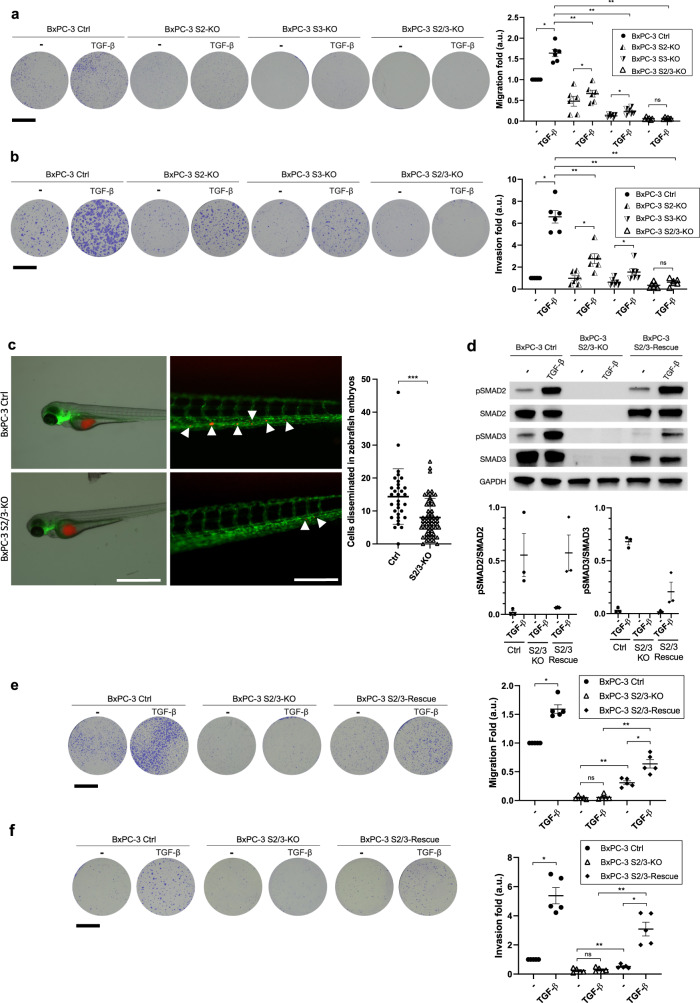
SMAD2 and SMAD3 are crucial in the migratory and invasive potential of BxPC-3 (SMAD4-negative) cells in response to TGF-β. **a** Images of a transwell migration assay with control (Ctrl), S2-KO, S3-KO, and S2/3-KO genetically engineered BxPC-3 cells cultured for 24 h in the presence or absence of TGF-β. Scale bar = 3 mm. Graphs represent mean values +/– SEM (*n* = 6). One-tailed Wilcoxon/Mann–Whitney test; **p-value < 0.01; *p-value < 0.05; *ns*, not significant. Scale bar = 3 mm. **b** Images of a transwell invasion assay with control (Ctrl), S2-KO, S3-KO, and S2/3-KO genetically engineered BxPC-3 cells cultured for 72 h in the presence or absence of TGF-β. Scale bar = 3 mm. Invasion was quantified as the mean value of six independent experiments and represented as a graph. Mean values +/– SEM are represented. One-tailed Wilcoxon/Mann–Whitney test; **p-value < 0.01; *p-value < 0.05; *ns*, not significant. **c** Images (left panel) of zebrafish embryos (fli:GFP) grafted with pre-stained control or S2/3-KO BxPC-3 cells (in red). Zebrafish embryos were incubated at 28 °C for 24 h after engraftment, and then imaged on an EVOS Cell Imaging System microscope. White arrows point toward disseminated cells (in red). Scale bar (left-hand images) = 1 mm. Scale bar (right-hand images) = 0.25 mm. Right panel: quantification of invaded metastatic cells per embryo, performed 24 h post-engraftment. Mean values (horizontal bars) +/– SD are represented. One-tailed Wilcoxon/Mann–Whitney test; ***p-value < 0.001. **d** Immunoblot of phospho-SMAD2 (pSMAD2), SMAD2, phospho-SMAD3 (pSMAD3), SMAD3 and GAPDH on control (BxPC-3 Ctrl), S2/3-knockout (BxPC-3 S2/3-KO) and S2/3-Rescue (BxPC-3 S2/3-Rescue) cells treated or not with TGF-β for 1 h. One representative image out of 3 independent experiments is shown. pSMAD/SMAD ratios were quantified from 3 independent experiments and represented as graphs of mean values +/– SD. **e** Images of a transwell migration assay with control, S2/3-KO, and S2/3-Rescue BxPC-3 cells cultured for 24 h in the presence or absence of TGF-β. Scale bar = 3 mm. Graphs represent mean values +/– SEM of five independent experiments. One-tailed Wilcoxon/Mann–Whitney test; **p-value < 0.01; *p-value < 0,05; *ns*, not significant. **f** Images of a transwell invasion assay with control, S2/3-KO, and S2/3-Rescue BxPC-3 cells cultured for 72 h in the presence or absence of TGF-β. Scale bar = 3 mm. Graphs represent mean values +/– SEM of five independent experiments. One-tailed Wilcoxon/Mann–Whitney test; **p-value < 0.01; *p-value < 0,05; *ns*, not significant.

Since TGF-β is known to facilitate tumor progression and aggressiveness, notably by stimulating cell migration, we analyzed the migratory potential of controls and S2/3-KO BxPC-3 cells in the presence or absence of TGF-β in a transwell migration assay. Control BxPC-3 cells displayed significant migratory properties, which were potentiated by TGF-β (1.7-fold), compared to S2/3-KO with negligible motility both in the presence and absence of TGF-β (Fig. 2a). Of note, differences between control and S2/3-KO cells in normal culture conditions could result notably from the presence of TGF-β in the medium as measured by ELISA assay, which could result from autocrine production. Single S2-KO and S3-KO cells displayed a significant decrease in migratory properties, but to a lesser extent than the double S2/3-KO cells, suggesting that both proteins take part in migratory processes (Fig. 2a). RepSox completely abolished TGF-β-induced migration of control BxPC-3 cells, showing that this phenotype relies on TβRI activation (Fig. S2a). Similar results were obtained using S2-KD, S3-KD, S2/3-KD BxPC-3 (Fig. S2b, c) and S2/3-KD Capan-1 cells (Fig. S2d). Thus, loss of SMAD2/3 expression in a cellular context devoid of SMAD4 alters their propensity to migrate in response to TGF-β.

Invasive potential, a hallmark of malignant cell aggressiveness, contributes to their dissemination. Along this line, we observed that SMAD2/3 depletion reduced the spreading of engrafted tumor cells over chicken chorioallantoic membranes (CAMs) (Fig. S2e). TGF-β promotes invasion via a complex transcriptional reprogramming that leads to cytoskeletal rearrangements and extracellular matrix (ECM) degradation. In vitro, the role played by SMAD2/3 in the invasive properties of BxPC-3 cells in response to TGF-β was then investigated using a Matrigel™-coated transwell assay (Fig. 2b). Following 72 h of TGF-β treatment, control BxPC-3 cells showed a 6.5-fold increase in cell invasion compared to untreated cells. Inversely, no increase was observed upon TGF-β treatment in S2/3-KO BxPC-3 cells, which retained a very low invasive potential. Single S2-KO and S3-KO cells displayed an intermediate phenotype, with a significant decrease in invasive properties, but to a lesser extent than S2/3-KO cells (Fig. 2b). RepSox completely abolished TGF-β-induced invasion of control BxPC-3 cells, showing that this phenotype also relies on TβRI activation (Fig. S2f). Similar results were obtained with S2/3-KD BxPC-3 cells (Fig. S2g, h) and S2/3-KD Capan-1 cells (Fig. S2i). We confirmed those in vitro results using a zebrafish embryo model that was previously reported to be suitable for determining invasive properties of tumor cells^34^. Control and S2/3-KO BxPC-3 cells pre-stained with a fluorescent lipophilic dye (DiD) were injected into the yolk sac of (fli:GFP) Casper zebrafish embryos. Migratory potential was assessed 24 h post-injection, by quantifying the number of tumor cells that reached the tail through the circulatory system, mimicking metastases spreading. S2/3-KO BxPC-3 cells displayed a lower migratory rate compared to control BxPC-3 cells, since we observed 1.6-times more cells in embryo tails engrafted with control versus S2/3-KO BxPC-3 cells (Fig. 2c).

To investigate whether the acquisition of migratory and invasive properties in a SMAD4-negative context relied on SMAD2/3, we stably rescued SMAD2 and SMAD3 expression in S2/3-KO BxPC-3 cells (BxPC-3 S2/3-Rescue), reaching 100% of the initial level for SMAD2 and about 30% for SMAD3 (Fig. 2d). As expected from this partial rescue, SMAD2/3 re-expression was sufficient to significantly increase both basal and TGF-β-induced migration (Fig. 2e) and invasion (Fig. 2f), as illustrated by a 7-fold increase in migration and invasion, compared to S2/3-KO BxPC-3 cells.

Collectively, these results demonstrate that in a cellular context devoid of SMAD4, SMAD2 and SMAD3 are necessary to transduce TGF-β-driven cell migratory, and invasive properties.

### TGF-β induces a SMAD2/SMAD3-dependent pro-migratory transcriptional program leading to a collective migration phenotype in SMAD4-negative pancreatic cancer cells

To define whether phenotypic differences between control and S2/3-KO BxPC-3 cells resulted from the activation of a peculiar transcriptional program by SMAD2/3 in the absence of SMAD4, we performed RNA-sequencing analysis (RNA-Seq). Triplicate RNAseq raw data from control and S2/3-KO BxPC-3 cells, treated or not with TGF-β (1 h and 24 h) were normalized using a standard pipeline.

**Fig. 3:**
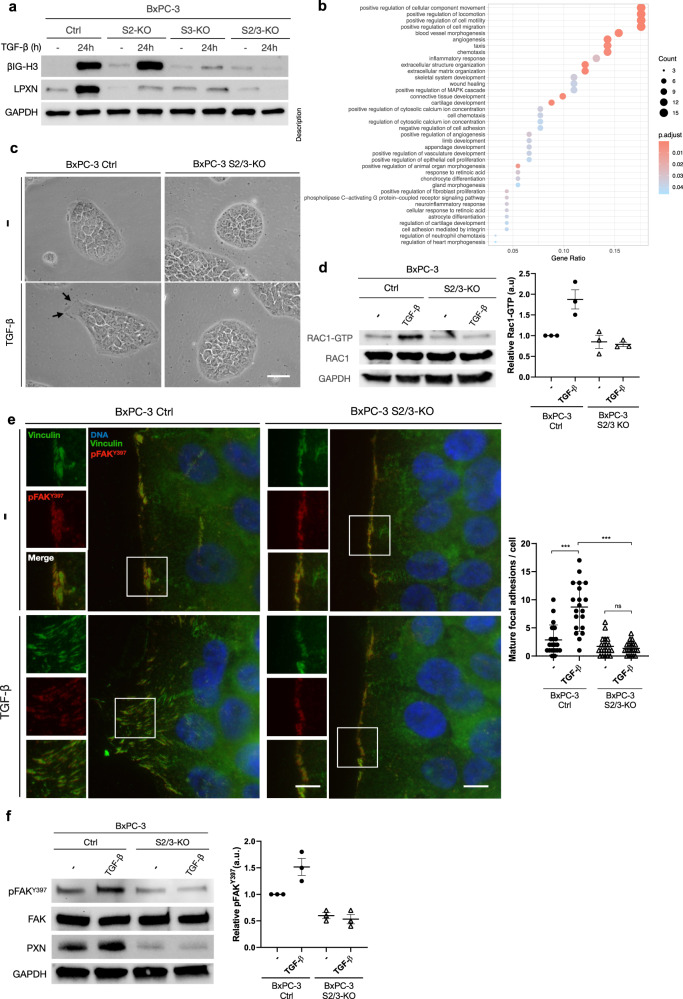
SMAD2/3 initiate a collective migration transcriptional program in response of TGF-β in a SMAD4-null PDAC context. **a** Immunoblot analysis of βIG-H3, LPXN and GAPDH on control (Ctrl), S2-KO, S3-KO, and S2/3-KO BxPC-3 cells treated or not with TGF-β for 24 h. One representative image out of 3 independent experiments is shown. **b** Scatterplot showing the top 25 enriched biological pathways using Gene Ontology (GO) database based on a list of 144 differentially expressed genes between control and S2/3-KO that are specific to the TGF-β treatment response at 24 h. Dots are colored according to the adjusted statistical probabilities (FDR) and sized by the count number of genes matching the biological process. Gene *ratio* corresponds to the relative number of input genes and known associated genes of the tested biological pathway. **c** Bright field microscopy images of control and S2/3-KO BxPC-3 cells after TGF-β treatment. Cells were treated for 5 days, to allow potential EMT transition. One representative image out of 3 independent experiments is shown. Black arrows indicate the presence of large lamellipodia detectable only in control cells in the presence of TGF-β. Scale bar = 30 µm. **d** Immunoblot of RAC-1-GTP, RAC-1, and GAPDH on control and S2/3-KO BxPC-3 cells treated or not with TGF-β for 15 min. Graphs represent RAC1-GTP mean values +/– SD (*n* = 3). **e** Images of Vinculin and phospho-Focal Adhesion Kinase (pFAK^Y397^) immunofluorescence performed on control and S2/3-KO BxPC-3 cells treated or not with TGF-β for 8 h, to avoid complete wound closure. Insets represent enlarged views of regions to visualize focal adhesion (FA) in leader cells. Nuclei were counterstained in blue with DAPI. Scale bar (left-hand images) = 2.5 µm. Scale bar (enlarged merge images) = 5 µm. Mean number of mature focal adhesions per cell with enlarged lamellipodia are represented as a graph. Variance is indicated as SD. One-tailed Wilcoxon/Mann–Whitney test; ***p-value < 0.001; *ns*, not significant. **f** Immunoblot of Paxillin (PXN), phospho-Focal Adhesion Kinase (pFAK^Y397^), Focal Adhesion Kinase (FAK), and GAPDH on control and S2/3-KO BxPC-3 cells treated or not with TGF-β for 24 h. One representative image out of 3 independent experiments is shown. Ratios of pFAK^Y397^/FAK were quantified from 3 independent experiments and represented as graphs of mean values +/– SD.

We first use differential analyses and a Venn diagram (Fig. S3a) to define the subset of genes differentially expressed in control and S2/3-KO BxPC-3 cells solely upon TGF-β treatment: 199 genes were identified as specifically differentially expressed 1 h post-treatment, and this number reached 964 at 24 h (Fig. S3b and Table S1). We confirmed these RNA-seq results by reverse transcription-quantitative polymerase chain reaction (RT-qPCR) and immunoblot for some SMAD2/3 putative target randomly chosen, and showed that *FGF1, ITGB6, LPXN* and *TGFβI* expression was upregulated after 24 h of TGF-β treatment in control BxPC-3, but not in S2/3-KO BxPC-3 cells (Fig. S3c). TGF-β-induced LPXN and βIG-H3 expression was also blocked in S2/3-KO at the protein level, as shown in Fig. 3a. Interestingly, a similar blockade was observed solely for βIG-H3 in SMAD3, but not in SMAD2, single-KO cells, suggesting that although complementary, SMAD2 and SMAD3 may have specific effects in the absence of SMAD4. Importantly, re-expression of SMAD2/3 in S2/3-rescue BxPC-3 cells was sufficient to restore TGF-β-induced regulation of these genes (Fig. S3d). Thus, SMAD2/3 likely drive the expression of a specific transcriptional program in the absence of SMAD4.

Non-canonical pathways induced by TGF-β can also induce transcriptomic changes. Thus, to precisely delineate SMAD2/3 target genes in the absence of SMAD4, we decided to focus -among the 964 genes described above-, on the 144 genes differentially expressed after 24 h exposure to TGF-β in control BxPC-3 cells, but not in S2/3-KO BxPC-3 cells (Table S2). Indeed, these genes likely correspond to SMAD2/3-specific targets in a SMAD4-deficient context and could also reflect the different phenotypic responses between control and S2/3-KO BxPC-3 cells observed upon TGF-β treatment. We performed Gene Set Enrichment Analyses using pathways from GO on this panel of 144 genes. Interestingly, we identified an over-representation of genes involved in the induction of migration and motility (Fig. 3b), which may explain the phenotypic migratory differences observed in the context of SMAD2/3 expression.

Of note, among the genes that were inactivated in control cells upon TGF-β treatment, we identified several epithelial cell adhesion/junction molecules, such as KRT13 and 15, but not E-Cadherin or β-Catenin, the expression of which remained unchanged upon TGF-β stimulation. Although we cannot exclude that non-transcriptional mechanisms could modulate the activity of these proteins, as previously shown for E-Cadherin^35^, we reciprocally observed a gain of some mesenchymal markers such as FN1. Such persistence in epithelial and cell/cell junction markers (E-Cadherin, β-Catenin), with the simultaneous extinction of others (KRT) and induction of mesenchymal markers (FN1) is one of the characteristics of collective migration^35–37^. Collective migration is a process whereby cells move as a cohesive multicellular unit and retain strong cell–cell interactions with specialized “leader cells” located at the invasive front^38^. In those leader cells, migratory properties were shown to rely on actin cytoskeletal remodeling through activation of a FAK (focal adhesion kinase)-Rac1 signaling axis. Interestingly, using KEGG pathway analysis, we identified a specific enrichment in genes involved in the regulation of focal adhesions (FA, hsa04510), which are complex membrane structures playing a major role in cell spreading, lamellipodia extension and cell migration^39^. More precisely, the expression of molecules acting upstream of FAK, such as COL1A1, FN1, ITGB6, FGF1, LAMC2 or EFNA2 were all significantly upregulated in control BxPC-3 cells 24 h after TGF-β treatment, but not in S2/3-KO BxPC-3 cells.

Such an increase in the expression of FAK activating molecules provides a credible scenario to explain how TGF-β could confer pro-migratory and pro-invasive properties to SMAD4-deficient cells via the activation of a SMAD2/3-dependent transcriptional program notably modulating cell adhesion properties and cytoskeleton dynamics.

We then wondered whether migratory properties observed in control but not in S2/3-KO BxPC-3 cells upon TGF-β treatment could result from the induction of collective migration. Since TGF-β is a well-known EMT-promoting factor, we first excluded that decreased motility of S2/3-KO BxPC-3 cells could be due to a failure to engage in such a process in response to TGFβ. As shown in Fig. S3e, we did not observe a global switch towards the expression of EMT-mesenchymal markers such as Fibronectin, N-Cadherin, Vimentin, ZEB1 or Snail/Slug in response to TGF-β, contrary to MCF10-A cells, which are known to engage a full-EMT in response to TGF-β^40^. Moreover, upon TGF-β treatment, control BxPC-3 cells remained in a relative epithelial state, with only discrete changes of a few epithelial markers, while others remained expressed alongside mesenchymal ones^38,41^. We then assessed morphological changes in both control BxPC-3 and S2/3-KO BxPC-3 cells cultured for 5 days in the presence or absence of TGF-β (Fig. 3c and Supplementary Movie 1). In the presence of TGF-β, control cells remained cohesive with an epithelial shape, without clear morphological evidence of EMT. However, we observed slight changes at the leading cells localized at the migration front, with the emergence of large lamellipodia, which were absent in S2/3-KO BxPC-3 cells. Similar results were obtained with Capan-1 cell line, with cells migrating in groups (Fig. S3f and Supplemental Movie 2).

Small GTPases, such as Rac1 and Rho-A are well described as mediators of migration through cytoskeletal remodeling and have critical roles in the invasive and metastatic behavior of cancer cells^42^. Rac1-GTP is notably involved in lamellipodia extension and focal adhesions during collective migration^43^. We thus investigated the activation of Rac-1 in both cell lines treated or not with TGF-β (Fig. 3d). Total Rac1 levels remained unchanged in both cell lines, whereas activated Rac1-GTP-bound levels increased only in control BxPC-3 cells treated with TGF-β (1.8-fold). Thus, Rac1 activation upon TGF-β treatment relies on SMAD2/3 expression in a SMAD4-deficient context.

To further determine whether the protrusive cells with enlarged lamellipodia were effectively “leader cells” and considering our RNAseq data, we used immunofluorescence microscopy to evaluate the expression of focal adhesion (FA) mediators and components. During cell migration, vinculin, an actin-binding cytoskeletal protein, is recruited to FA and co-localizes with other critical effectors of cell migration such as phosphorylated active FAK (pFAK^Y397^) or Paxillin (PXN) to control FA turnover^44–47^. We performed co-IF experiments to detect vinculin and pFAK^Y397^ in control and S2/3-KO BxPC-3 cells treated with TGF-β (Fig. 3e). In control cells, vinculin and pFAK^Y397^ co-localized in FA at the edge of the cell monolayer. The staining pattern switched from a dotted pattern in the absence of TGF-β to a “claw-like” pattern in the enlarged lamellipodia in response to TGF-β treatment, indicative of FA maturation, whereas it remained unchanged in S2/3-KO BxPC-3 cells, maintaining FA in a nascent state. Immunoblot analysis of Paxillin and p-FAK^Y397^ showed that TGF-β potentiated the expression of Paxillin and the phosphorylation of FAK in control but not in S2/3-KO BxPC-3 cells (Fig. 3f), strengthening the involvement of SMAD2/3 in TGF-β-induced focal adhesion maturation in the absence of SMAD4. Similar results were obtained with Capan-1 cells (Fig. S3g). We thus investigated whether a Rho-A inhibitor (Rhosin), a Rac1 inhibitor (NSC 23766), a pFAK^Y397^ inhibitor (Defactinib) or the calcium-chelating agent EGTA, which interferes with cell–cell junctions by disrupting cadherin-cadherin homotypic interactions, affected BxPC-3 cell migration in response to TGF-β. As shown in Fig. S3h, all inhibitors drastically decrease the migratory propensities of cells, but only Defactinib significantly reduced TGF-β-induced migration, further emphasizing the crucial role of the FAK pathway in this process.

Hence, TGF-β-induced SMAD2/SMAD3-dependent migration in SMAD4-negative PDAC cells requires the activation of FAK, Rac cascade, as well as cell–cell adhesions, which are hallmarks of collective cell migration.

### In human SMAD4-negative-PDAC patients, activation of SMAD2 is a marker of aggressiveness

**Fig. 4:**
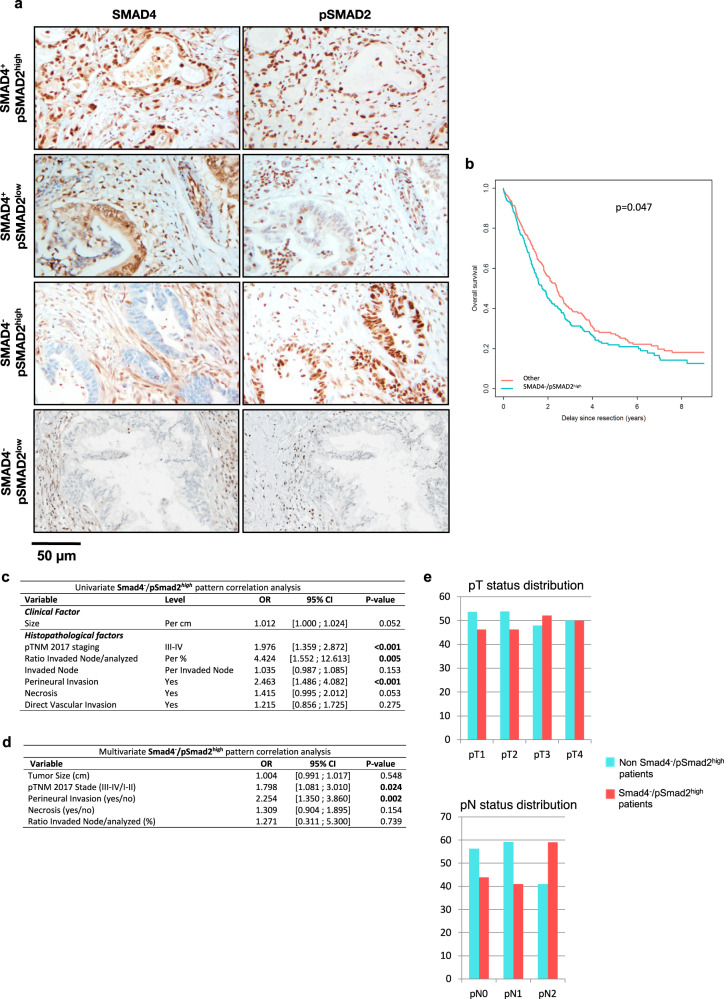
pSMAD2^high^/SMAD4^-^ pancreatic tumors are correlated with increased aggressiveness in vivo. **a** Immunohistochemistry for SMAD4 and pSMAD2 on 506 clinically annotated resected primary human PDAC. The tumors were separated into four groups according to SMAD4 and pSMAD2 status (SMAD4^+^/pSMAD2^high^, SMAD4^+^/pSMAD2^low^, SMAD4^-^/pSMAD2^high^, SMAD4^-^/pSMAD2^low^). **b** Survival curve of patients with SMAD4^-^/pSMAD2^high^ PDAC compared to pooled three other groups. **c** Univariate analysis of the correlation between the SMAD4^-^/pSMAD2^high^ tumor pattern and aggressiveness/histopathological features. **d** Multivariate SMAD4^-^/pSMAD2^high^ pattern correlation analysis. **e** pT and pN status distribution for SMAD4^-^/pSMAD2^high^ tumors and non-SMAD4^-^/pSMAD2^high^ tumors.

To assess the clinical relevance of the oncogenic role of SMAD2/3 in the absence of SMAD4, we analyzed 506 proprietary resected primary human PDAC samples that we clinically and morphologically annotated. Loss of SMAD4 occurs in 50% of the PDAC cases^48^, but the status of SMAD2/3 phosphorylation—i.e., of their activation status—has never been explored in situ. No antibody specifically recognizes pSMAD3 in immunohistochemistry (IHC), both in literature and the multiple tests we realized. However, we successfully succeed in setting up a robust and reproducible protocol to assess pSMAD2 and SMAD4 status by IHC. We verified the specificity of these antibodies on placenta (positive control) and lymph node (negative control) (Fig. S4a). We analyzed the expression of pSMAD2 and SMAD4 on serial sections of 506 primary human PDACs and classified these tumors into four groups (SMAD4^+^/pSMAD2^high^, SMAD4^+^/pSMAD2^low^, SMAD4^−^/pSMAD2^high^, SMAD4^-^/pSMAD2^low^) (Figs. 4a and S4b). Consistent with the literature, 253 samples (50% of all samples) stained negative for SMAD4. Importantly, both SMAD4 (when positive) and pSMAD2 were clearly nuclear. Among these, 240 cases (47.4% of all samples) showed high pSMAD2 expression and stained negative for SMAD4 (SMAD4^-^/pSMAD2^high^ tumors), while the rest of the cases were split into SMAD4^+^/pSMAD2^high^ (*n* = 241, 47.6%), SMAD4^+^/pSMAD2^low^ (*n* = 12, 2.4%) and SMAD4^-^/pSMAD2^low^ (*n* = 13, 2.6%) tumors (Fig. S4b, c).

When we explored the overall survival based only on SMAD4 expression, no statistical difference between the SMAD4^-^ and SMAD4^+^ groups was unveiled (1.9 [1.6, 2.3] versus 2.4 [1.9, 2.7], *p* = 0.14) (Fig. S4c, right upper panel), as previously reported by others on North American or European patients series^49,50^. Similarly, pSMAD2 expression alone did not discriminate groups (2.4 [2.1, NA] versus 2.0 [1.8, 2.4], *p* = 0.23) (Fig. S4c, right lower panel). On the contrary, SMAD4^-^/pSMAD2^high^ tumors showed a trend towards a worse prognosis, and SMAD4^-^/pSMAD2^low^ towards a better prognosis (Fig. S4c, left upper panel). Multivariate analysis includes the model of pTNM staging, which encompasses most on the prognostic factors, as shown by non-significance of robust prognostic criteria such as neural invasion and resection quality (Fig. S4d). However, the prognostic value of SMAD2 activation in settings of SMAD4 deficiency was confirmed in univariate analysis, since patients with SMAD4^-^/pSMAD2^high^ tumors had a poorer overall survival than patients with other patterns of expression (*p* = 0.047) (Fig. 4b). The patients with SMAD4^-^/pSMAD2^high^ tumors displayed a reduction of their median overall survival of 0.7 years, corresponding to approximately 8 months (Fig. 4b). This analysis is consistent with our hypothesis that prognosis is not driven solely by SMAD4, but that the combination of SMAD4^-^ and pSMAD2 protein expression may be considered of prognostic value.

To better characterize the SMAD4^-^/pSMAD2^high^ group of tumors *vs* pooled other groups and explain the worse overall survival, we analyzed the correlation between pSMAD2/SMAD4 expression (by IHC) and other known and validated histo-prognostic factors. In univariate analyses, SMAD4^-^/pSMAD2^high^ was associated with increased perineural invasion, a higher *ratio* of invaded/analyzed lymph nodes and higher TNM stage (*p* < 0.001, *p* < 0.005, and *p* < 0.001, respectively), whereas only perineural invasion and higher TNM stage remained statistically associated with SMAD4^−^/pSMAd2^high^ in multivariate analysis (Fig. 4c, d). As higher TNM stages correspond to more aggressive tumors, we investigated whether SMAD4^-^/pSMAD2^high^ tumors displayed more advanced local (T stage) or regional (N stage) aggressiveness. The distribution of tumors according to the T and N stages showed that the aggressiveness of these tumors could be driven by two different mechanisms. The first one is perineural invasion, which is twice as frequent in the SMAD4^-^/pSMAD2^high^ tumors (2.463 [1.486; 4.082], *p* < 0.001, Fig. 4c) and is consistent with higher T stages (Fig. 4e), reflecting the local invasion along the nerve sheet. The second mechanism corresponds to regional lymph node metastasis with a higher *ratio* of invaded/analyzed lymph nodes (OR: 4.424 [1.552; 12.613], *p* = 0.005, Fig. 4c), consistent with a higher number of N2 stage in SMAD4^-^/pSMAD2^high^ tumors (Fig. S4e). Taken together, our data show that SMAD2/3 TGF-β pathway activation is detectable in the absence of SMAD4 in human PDAC and is correlated with poorer survival, associated with the presence of aggressive features such as perineural invasion and lymph node metastasis.

This analysis is consistent with our hypothesis that prognosis is not driven solely by SMAD4, but that the combination of SMAD4^-^ and pSMAD2 protein expression may be considered of prognostic value.

## Discussion

In the present study, we provide evidence that the oncogenic properties of TGF-β in SMAD4-negative BxPC-3 cells are associated with the activation of a transcriptional program triggered by SMAD2 and SMAD3. This transcriptional program supports the activation of a Rho/Rac-dependent invasive migratory program related to “collective migration” involving vinculin and FAK. Finally, we explored in vivo the physiological relevance of SMAD2 activation in 506 human PDAC samples and evidenced that SMAD4-negative tumors with high levels of pSMAD2 were more aggressive and had a poorer prognosis.

Mechanistically, we demonstrated that control BxPC-3 cells migrate in clusters in response to TGF-β, only in presence of SMAD2/3, with no obvious morphological features of EMT and no or few changes in classical EMT molecular markers. TGF-β-driven tumor migration is generally associated with the capacity of TGF-β to induce EMT-associated single cell migration. However, it has previously been reported that cancer cells do not rely on the EMT process to gain in aggressiveness, and that EMT and migration can occur independently^16,26,51,52^. A recent study by David et al.^30^ even showed that TGF-β-induced EMT in SMAD4-positive PDAC is lethal by ultimately leading to cell apoptosis. In the present study, we observed that SMAD2/3 expression in a SMAD4-deficient context is associated with morphological, sub-cellular and genetic changes reminiscent of collective migration, which is an alternative migratory process in both development and cancer^41,53,54^.

Collective migration is an orchestrated cell movement relying on the dynamic assembly of complex multiproteic membrane structures known as focal adhesions (FA)^47,55^. FA, are large macromolecular assemblies that form at the leading edge of the cell in lamellipodia, and consist of integrins, kinases (such as FAK) and adapter/cytoskeletal proteins (such as vinculin)^56^. In addition, vinculin binds FAK, which is in turn able to regulate the activity of Rac, promoting the formation of new adhesions at the leading edge^57^. Previous studies have shown a crucial role for Rac1 in TGF-β/SMAD and non-SMAD-mediated cellular responses^58–60^. Here, our findings support that SMAD2/3 mediate oncogenic properties of TGFβ in the absence of SMAD4 in pancreatic tumor cells, by conferring them a collective migratory ability through modulation of Rho/Rac and FAK activities to form FA. As non-canonical TGF-β signaling and the SMAD-dependent BMP pathway were not affected, albeit slightly delayed, after TGF-β treatment in S2/3-KO BxPC-3 cells, we cannot exclude a synergistic role for these pathways with SMAD2/3 in the absence of SMAD4. Of note, aside from their relevance to study SMAD2/3 independently of SMAD4, our cellular models could then also be useful to further characterize the non-canonical pathways and associated crosstalks.

Gene set enrichment analyses performed on SMAD2/3-dependent target genes at 24 h of TGF-β treatment uncovered a biological signature reinforcing the crucial role of SMAD2/3 in migration and motility (e.g., locomotion, cell motility, cell migration, ECM organization…) such as *COL1A1*, *FN1*, *ITGB6*, *FGF1, LAMC2, EFNA2, MYL9 or ABLIM2*. Recently, an integrated transcriptome meta-analysis of pancreatic ductal adenocarcinoma identified 28 upregulated genes in PDAC compared to both adjacent and normal pancreatic tissues that may be used as promising prognostic and diagnostic biomarkers for PDAC^61^. Our results identified 3 of these 28 genes that were upregulated 24 h after exposure to TGF-β in control BxPC-3 cells, but not in S2/3-KO BxPC-3 cells (e.g*., ITGB6, LAMC2*, and *TGM2*). Interestingly, Zhuang et al. recently demonstrated that ITGB6 overexpression is significantly associated with upregulation of FA signaling pathways in pancreatic cancer^62^, and this gene was also recently included in a prognostic signature (comprising 9 genes) of pancreatic cancers by Wu and colleagues^63^. In addition, we observed that the transcriptional changes in the expression of genes such as *COL1A1, FN1, ITGB6, FGF1, MYL9 or ABLIM2*, which are known to modulate Rac1 activity or the organization of cytoskeleton, are all SMAD2/3-dependent (Table S2). Thus, we assumed that a SMAD2/3-dependent transcriptional switch upon TGF-β treatment is responsible for increased migratory properties of PDAC cells in the absence of SMAD4. Importantly, this SMAD4-independent activity of SMAD2/3 argues in favor of the critical contribution of other partners to their nuclear translocation, further emphasizing the complexity of this network^28,29^.

In human tumors, our findings show that activation of the SMAD2/3 TGF-β pathway persists in the absence of SMAD4 in a subset of patients. Although the characterization of SMAD3 phosphorylation level would have been interesting considering the major phenotypic role of this effector, we observed that the persistence of pSMAD2 in the absence of SMAD4 (i.e., SMAD4^-^/pSMAD2^high^ tumors) is associated with increased perineural invasion, lymph node metastasis, necrosis and poor prognosis. Of note, even though the pSMAD2 low-expression group is small, the significance of adding the 13 patients with pSMAD2 low/SMAD4—expression is statistically relevant, thereby arguing for the contribution of this parameter beside SMAD4 expression status. From our point of view, persistent pSMAD2 expression in the vast majority of PDAC (95%) supports the idea of a positive selection pressure for this factor associated with oncogenic effects. Our results are consistent with the association of perineural invasion and loss of SMAD4 reported previously^64,65^. The difference in overall survival of more than 8 months between Smad4^-^/pSmad2^high^ and other patients, corresponds to an approximate reduction of one third in post-resection overall survival, which is non-negligible for patients.

Then, our findings that SMAD4^-^/pSMAD2^high^ human PDAC are more aggressive, suggest that combined immunohistochemistry for SMAD4 and pSMAD2 may improve PDAC patient stratification and identify a poor prognostic subgroup. From a clinical point of view, several FAK inhibitors have entered clinical development, but so far, the use of these agents as monotherapy has been limited^66,67^. Our results show that FAK activation in PDAC is at least partly mediated by perverted SMAD2/3-TGF-β signaling in PDAC cells that have lost SMAD4. Thus, concomitant inhibition of FAK and TGF-β signaling using either TGF-β-RI kinase inhibitors (such as galunisertib) or monoclonal antibodies (such as frezolizumab) may act synergistically in pancreatic cancer. Finally, the identification of SMAD2/3-induced genes by RNAseq may provide new therapeutic targets for PDAC.

In conclusion, this work reveals that SMAD4 inactivation in PDAC not only results in a loss of SMAD4 tumor suppressive function but is concomitant to the oncogenic gain-of-function of SMAD2 and SMAD3. Further investigations are required to determine whether SMAD4-positive cancer cells, in which SMAD4 cytostatic and pro-apoptotic functions are active, also use SMAD2 and 3 as oncogenic factors. This could broaden the potential therapeutic options for SMAD2- and 3-dependent TGFβ target genes

## Methods

### Cell lines culture and treatment

The BxPC-3 (SMAD4 negative^68^, ATCC CRL-1687 human pancreatic adenocarcinoma cell line was maintained in RPMI-1640 medium supplemented with 10% fetal bovine serum (FBS) and 1% penicillin/streptomycin (P/S). BxPC-3 cells with targeted disruption of *SMAD2* and/or *SMAD3* genes (S2-KO, S3-KO, and S2/3-KO) were established using the CRISPR/Cas9 system. Plasmid construct for *SMAD2* CRISPR/Cas9, bearing the specific single-guide RNA targeting the exon 2 of *SMAD2* (GATGGAAGAAGTCAGCTGGT), the puromycin resistance and the hCas9 were stably transfected in the BxPC-3 cell line (2 µg). Plasmid construct for *SMAD3* CRISPR/Cas9 gene knockout, bearing the specific single-guide RNA targeting the exon 6 of *SMAD3* (GGAATGTCTCCCCGACGCGC) and the hygromycin resistance cassette were stably inserted in HEK293LTV cells to produce lentiviral particles. LV suspension was added in BxPC-3 medium during 12 h. Infected cells were selected with the antibiotic puromycin (1 µg/mL) and hygromycin (250 µg/mL) and tested for efficient gene knockout (KO) by immunoblot, immunofluorescence and flow-cytometry with specific SMAD2 and SMAD3 antibodies.

Plasmid construct for SMAD2/3-rescue was inserted into HEK293LTV cells to produce lentiviral particles. LV suspension was added in S2/3-KO BxPC-3 cell medium and cells selected with blasticidin (5 µg/mL).

Plasmids constructs for SMAD2 and SMAD3-knockdown (shRNA, sequences available upon request), harboring shRNA sequences designed against SMAD2 or SMAD3 antibiotic resistance cassette for selection, were inserted into HEK293LTV cells to produce lentiviral particles. LV suspension was added in BxPC-3 and Capan-1. Sh-S2/3-cells were selected with blasticidin (5 µg/mL) and hygromycin (250 µg/mL).

For all experiments, BxPC-3 and Capan-1 cells were seeded in RPMI or IMDM medium, respectively, supplemented with 10% fetal bovine serum (FBS) and 1% P/S (without puromycin), and they were serum starved (1% FBS medium) for 12 h prior to each experiment.

PANC-1 (SMAD4 positive, ATCC CRL-146) human pancreatic adenocarcinoma cell line were maintained in DMEM supplemented with 10% FBS and 1% P/S. MCF 10 A (ATCC CRL-10317) human breast cell line was maintained in MEMB supplemented with 10% fetal bovine serum (FBS) and 1% P/S. All cells were grown at 37 °C in a humidified atmosphere containing 5% CO_2_.

### Reagents

Recombinant human TGF-β1 (TGF-β1) was purchased from PeproTech (#100-21 B). RepSox (TβRI kinase activity inhibitor, #S7223) was from Selleckchem. 5-Fluorouracil (5-FU, #F6627) was from Sigma. The Rho inhibitor (Rhosin #5003) and the RAC1 inhibitor (NSC23766 #2161) were from Biotechne. Antibodies against SMAD2 (#5339), SMAD3 (#9523), phospho-SMAD2 (Ser465/467) (#3108), phospho-SMAD3 (Ser423/425) (#9520), SMAD2/3 (#8685), SMAD4 (#46535), RAC1 (#4651), βIG-H3 (#5601) and Leupaxin (#59309) were purchased from Cell Signaling. The anti-GAPDH antibody (#8245), anti-phospho-histone H3 (#14955), anti-Snail/Slug (#85936), SMAD1/5/9 (#80255) were from Abcam. Anti-Paxilin (PXN) (PA5-111334) and anti-pFAK^Y397^ (44-625 G) were from Invitrogen. Anti-vinculin (V9131), anti-FAK (#05-537) and anti-ZEB1 (#HPA027524) were from Sigma. ZO-1 (# 610966), E-cadherin (#610405), β-Catenin (#610154), Fibronectin (#610077) and N-Cadherin (#610920) primary antibodies were from BD Biosciences. Antibody against vimentin was from DAKO (#GA63061). HRP-coupled anti-rabbit secondary antibody were from Immuno Reagents (GtxRb-003-DHPRX). HRP-coupled anti-mouse secondary antibody were from Dako Cytomation (P0260). Culture media were obtained from GIBCO-Invitrogen. Nuclear and cytoplasmic extraction reagent (ThermoFischer NE/PER kit #78833) was used for cell fractionation assay before immunoblots. PAK1 PBD Anti-RAC1-GTP agarose beads (# STA-411) were from Cell Biolabs.

### Immunoblot analysis

For protein analysis, cells were washed once with cold phosphate-buffered saline (PBS), and lysed with RIPA buffer (50 mM Tris-HCl pH 7.4, 150 mM NaCl, 1 mM EDTA, 0.5% sodium deoxycholate, 0.1% SDS, 1% Nonidet) containing a protease and phosphatase inhibitor cocktail. After protein quantification, 10 to 50 μg of protein was used for total lysate samples. Samples were separated by SDS-polyacrylamide gel electrophoresis (SDS-PAGE) and detected by immunoblot (WB) using Amersham ECL Prime Detection Reagent (GE Healthcare).

### Immunofluorescence

To visualize the collective migration, cells were seeded in Ibidi 3-well removable chambers slides (#80381) at 1.5 × 10^5^ cells per well, in which a 2 wells culture-insert (Ibidi #80209) was added, defining a cell-free gap (wound). Upon confluency, cells were serum starved (1% FBS medium). The next day the culture-insert was removed, and the cells were treated or not with TGF-β (10 ng/mL) for 8 h.

Cells were fixed with 4% PFA (paraformaldehyde solution, Thermo Scientific) for 30 min, washed (PBS 1x), permeabilized with 0.1% Triton X-100 for 5 min, and blocked with PBS-5% FBS for 1 h. Labeling was performed by incubating cells for 1 h with specific antibodies for SMAD2/3, vinculin and phospho-FAK^Y397^. After three PBS 1x washes, cells were incubated with the specific secondary antibodies Alexa488-conjugated goat anti-mouse antibody (Life Technologies, A-11001), or Alexa594-conjugated goat anti-rabbit antibody (Life Technologies, A-11012). All antibodies were diluted in DakoReal Antibody Diluent (#S2022). Cells were washed three times with PBS 1x, and incubated with DAPI (Sigma d9542) for 5 min. Samples were mounted on microscope slides with DakoCytomation Fluorescent Mounting Medium. Images were acquired on an Upright epifluorescence microscope Zeiss AxioImager and treated with ImageJ Software.

### Flow cytometry

The proportion of SMAD2/3-positive cells in control and S2/3-KO BxPC-3 cells was assessed by flow-cytometry. Control and S2/3-KO BxPC-3 cells were detached, washed and resuspended with Flow-Cytometry Staining Buffer (eBioscience #00-4222-26). To assess cell viability, cells were stained with LIVE/DEAD Far Red Dead Cell solution (Invitrogen #L10120). Cells were fixed and permeabilized for 30 min with 1x FoxP3 Fixation/Permeabilization Buffer (eBioscience #00-5523-00), washed, resuspended in 1x Permeabilization Buffer (eBioscience #00-8333-56), then blocked with 5%-FBS Permeabilization Buffer for 30 min. Cells were then incubated with anti-SMAD2/3 antibody for 45 min, washed twice with 1x Permeabilization Buffer, incubated with anti-rabbit Alexa Fluor 488-conjugated antibody (Invitrogen A32731) for 30 min and washed twice with 1x Permeabilization Buffer. Samples were resuspended in Flow-Cytometry Staining Buffer before analysis with BD Canto II flow cytometer. Results were interpreted with BD DIVA Software.

### Migration assays

Transwell migration assay was used to assess the migration capacity of the cells (transwell assay, Corning #353097) followed by an immunofluorescence (IF) assay. 5 × 10^4^ cells were seeded in the insert chamber (8 µm pore size) and allowed to attach for at least 3 h in 0% FBS medium. Cells were then pre-treated with the anti-proliferative compound 5-Fluorouracil (5-FU, 8 µM) for 1 h and stimulated or not (Untreated) with TGF-β (10 ng/mL) for 24 h. If indicated, cells were also pre-treated 1 h either with RepSox (TβR1 kinase activity inhibitor), Rac Inhibitor (50 µM), Rho inhibitor (30 µM), phosphor-FAK inhibitor (1 µM) and EGTA (0.3 mM), then treated or not (untreated) with TGF-β (10 ng/mL) for 24 h.

Cells were then washed with PBS1x, methanol-fixed for 15 min and stained with crystal violet (0.1%) for 20 min. The quantification of cell migration was determined using ImageJ software.

### Invasion assays

Boyden Chambers with inserts coated with growth factor-reduced Matrigel (ECM Gel from Engelbreth-Holm-Swarm murine sarcoma, Corning #354230) were used to assess cell invasion. Briefly, 5 × 10^4^ cells per insert were resuspended in 100 µL of matrigel (5 mg/mL) and carefully seeded on top of each insert. Matrigel was left at 37 °C 1 h in order to allow solidification. Next, 700 µL of 10% FBS medium was added to the lower chamber and 300 µL of 0% FBS medium was added to the insert. The specific treatments were then applied to the insert upper chamber (5-Fluorouracil 8 µM to all wells, and TGF-β 10 ng/mL only to treated conditions). After 72 h, cells were washed with PBS 1x, methanol-fixed for 15 min and stained with crystal violet (0.1%) for 20 min. The quantification of cell invasion was determined using ImageJ software.

### Reverse transcription-quantitative polymerase chain reaction (RT-qPCR)

TGF-β target gene mRNA expression in BxPC-3 cells was assessed by RT-qPCR. Briefly, total RNA was isolated from cells treated or not (Untreated) with TGF-β (10 ng/mL) using RNAeasy Plus Mini Kit (Qiagen #74136), according to the manufacturer’s instructions. One microgram of total RNA were used for cDNA synthesis using random primers (Invitrogen #48190011), and Superscript RT (Invitrogen #18064014). qPCR was performed using the SYBR Fast SYBR Green Master Mix (Applied Biosystems #4385612) and the specific human primers listed in the Table S3. Data were analyzed using the 2^–ΔΔCt^ method to gene examine relative expression, presented as the fold-change over control and normalized to GAPDH.

### RNA sequencing

Material for RNA sequencing (RNA-Seq) was generated from control and S2/3-KO BxPC-3 cells treated or not with TGF-β (10 ng/mL) for either 1 h or 24 h. RNA was isolated, quantified and integrity was measured using an RNA assay kit on a 2100 BioAnalyzer (Agilent, RIN > 7). The samples were then subjected to RNA sequencing (Illumina Novaseq^TM^6000, Paired End). RNA-Seq data have been deposited in Gene Expression Omnibus (GEO, GSE178714).

#### Alignment and quantification

After careful quality controls, gene expression was quantified using Salmon (v1.1.0)^69^ and the annotation of known genes from Gencode v33^70^.

#### Quality controls

Using the raw expression matrix, we estimated the number of detected genes as the number of genes with at least 5 counts. Principal component analysis (PCA) was done with the plotPCA function of the DESeq2 R package^71^.

#### Gene expression and normalization

Unless otherwise specified, the analyses were performed using R (v 3.6.1) and illustrations produced with the ggplot2^72^ and ggpubr packages. Raw expression data were normalized to their log transcripts per million (logTPM) values before clustering and visualization.

#### Differential expression analysis

Starting from raw counts, we used the R package DESeq2 (v1.26) to perform the differential expression analysis. We performed four sets of differential analyses to compare gene expression levels between conditions (WT or S2/3-KO) with or without treatment after 1 h or 24 h. The design was set as ~replicate + condition. Differential expression was tested using the Wald test, and *p*-values were corrected with the Benjamini–Hochberg method. Genes were considered as significantly differentially expressed if their absolute log2 fold-change was above 1 with an adjusted *p*-value < 0.05.

#### Heatmap visualization

The top 50 genes were selected from the differential analyses based on their adjusted *p*-value < 0.05 after 1 h or 24 h TGF-ß treatment between conditions. Their expression value was then retrieved from the normalized matrix described before. Samples and genes were clustered using a Euclidean distance metric and complete linkage. Data were scaled by genes and heatmaps where then produced using GraphPad Software 8.0^72^.

#### Pathway enrichment analysis

To test the pathway enrichment of a list of genes, we used the entrez IDs as input and the R packages clusterProfiler (v 3.8.1)^73^ and org.Hs.eg.db (v 3.5.0)^74^. org.Hs.eg.db: Genome wide annotation for Human) (v 1.24). We tested the list of genes against pathways from msigdb GO. We selected pathways with an adjusted *p*-value < 0.01 (Benjamini–Hochberg method) and a *q*-value < 0.05.

#### Raw data repository

The raw RNA-seq data for this manuscript are available at GEO (GSE178714).

### Chicken chorioallantoic membrane (CAM) assay

On day 1 of embryonic development, fertilized chick eggs (*Gallus gallus domesticus*, EARL Morizeau) were incubated at 38 °C and a relative humidity of 80%. After 3 days, a window was cut in the shell. On day 11, 2 × 10^6^ of control or S2/3-KO BxPC-3 cells, resuspended in 100 µL of growth factor-reduced Matrigel, were inoculated on chorioallantoic membrane. Finally, after 17 days of development, chicken embryos were euthanized, and tumors were imaged. Their surfaces were determined using ImageJ software. All experiments were performed in accordance with relevant guidelines and regulations of animal ethics committee (Authorization APAFIS #33373; accreditation of laboratory animal care by ACCèS, CLB Lyon).

### Zebrafish embryo xenografts

(Fli:gfp) CASPER zebrafish (*Danio rerio*) embryos were raised at the Zebrafish Facility (IGFL, ENS Lyon). Prior to injection, 2 × 10^6^ control and S2/3-KO BxPC-3 cells (pre-stained with lipophilic DiD (ThermoFisher Scientific, V22889) for 20 min at 37 °C) were resuspended in 30 µL of PBS. Forty-eight hours post-fecundation, zebrafish embryos were dechorionated, anaesthetized with tricaine (Sigma-Aldrich, E10521) and injected with approximately 20 nL of either DiD-stained control or S2/3-KO BxPC-3 cells in the yolk sac. Embryos were further incubated at 28 °C for 24 h in E3 medium. In order to quantify cells that disseminated from the tumors to the tail, embryos were anaesthetized with tricaine with EVOS Cell Imaging System.

### Proliferation and apoptosis analysis

Control and S2/3-KO BxPC-3 cells were infected with the Nuclight lentivirus reagents (Sartorius #4625) in order to stained nucleus in red. 5 × 10^4^ cells were seeded in 48-wells plate. The next day, cells were serum starved (1% FBS medium), treated or not with TGF-β and cleaved-Caspase 3/7 Green dye (Sartorius #4440) was added in the culture medium to follow apoptosis. Images were acquired and analyzed for 72 h with an Incucyte ZOOM Imaging System (Essen Bioscience).

### Human PDAC cohort

All the samples from patients who had undergone surgery in the Hospices Civils de Lyon between the 1st of January 2004 and the 31st of December 2017 for a pancreatic ductal adenocarcinoma were included in the cohort. The clinical data were retrieved from the common medical files of each patient. The histopathological slides were reviewed by two pathologists including one expert in pancreatic diseases, blindfolded to the clinical and immunohistochemical data, to assess aggressiveness features. The review also allowed clinicians to select the most representative formalin-fixed paraffin-embedded (FFPE) sample in order to carry out immunohistochemical analyses.

### Immunohistochemical analysis of human PDAC samples

Two histological slides were selected to perform immunohistochemical staining (Ventana Ultra Benchmark) for SMAD4 (Abcam ab228205, clone SP306, prediluted kit) and pSMAD2 (Cell Signaling #3108, clone 138D4, [S465/467], dilution: 1/100). The two antibodies were revealed with peroxidase reaction (OptiView DAB Detection Kit, Roche). A positive control was present on each slide for SMAD4 and pSMAD2 (stroma reaction and native acinar pancreatic tissue). For SMAD4, an external negative control was part of the batch for each immunohistochemical analysis. SMAD4 and pSMAD2 staining was reviewed by two pathologists including one expert in pancreatic diseases, blindfolded to the clinical and morphological data. SMAD4 status was lost or conserved. SMAD4 loss was defined by the absence of nuclear staining, while its conservation was assessed based on the presence of nuclear staining. Based on previous study that correlated immunohistochemical and genetic pattern of SMAD4 alteration^75^, the tumor was to present a loss of SMAD4 if in the same tumor, two clones, one with loss and one with conservation of SMAD4 expression, were present. pSMAD2 status was high or low. To determine the pSMAD2 status, the signal intensity was compared to the signal intensity observed in the fibroblastic cells and the acinar pancreatic tissue (lower or higher).

### Statistics and reproducibility

For in vitro experiments, one-tailed Wilcoxon/Mann–Whitney tests were used to calculate statistical significance. *p*-value < 0.05 (*) < 0.01 (**),  < 0.001 (***) was considered as significant. Sample size and replicates are stated in corresponding in the corresponding figure legends. The survival curve for human resected samples was established based on the entire human PDAC cohort diagnosed following the WHO Classification of the digestive system (Bosman, 2010, book, ISBN: 9789283224327) and the log rank test was used, and the mean overall survival was given with a 95% confidence interval. A *p*-value < 0.05 was considered to be significant. For human overall survival analysis, the Cox model was used. For correlation with SMAD4^-^/pSMAD2^high^ pattern analysis, the odds *ratio* was calculated with logistic regression using a 95% confidence interval. A *p*-value < 0.05 was considered to be significant.

### Reporting summary

Further information on research design is available in the Nature Research Reporting Summary linked to this article.

## Supplementary information

Supplementary Information

Description of Additional Supplementary Files

Supplemental Movie 1

Supplemental Movie 2

Supplementary Data 1

Reporting Summary

## Data Availability

All data generated or analyzed during the study are included in this published article. Unedited blots are included as Fig. S5. The source data behind the graphs in the paper are provided as Supplementary Data 1. The RNA-Seq data have been deposited in the Gene Expression Omnibus database (GSE178714). Requests for material should be made to the corresponding authors.
