# Supplementary material for: SMAD2/3 mediate oncogenic effects of TGF-β in the absence of SMAD4
Source: Commun Biol. 2022 Oct 7;5:1068. doi: 10.1038/s42003-022-03994-6 (PMC9546935; doi:10.1038/s42003-022-03994-6)
Supplement: Supplementary file 1 — Supplementary Information [file 42003_2022_3994_MOESM1_ESM.pdf]

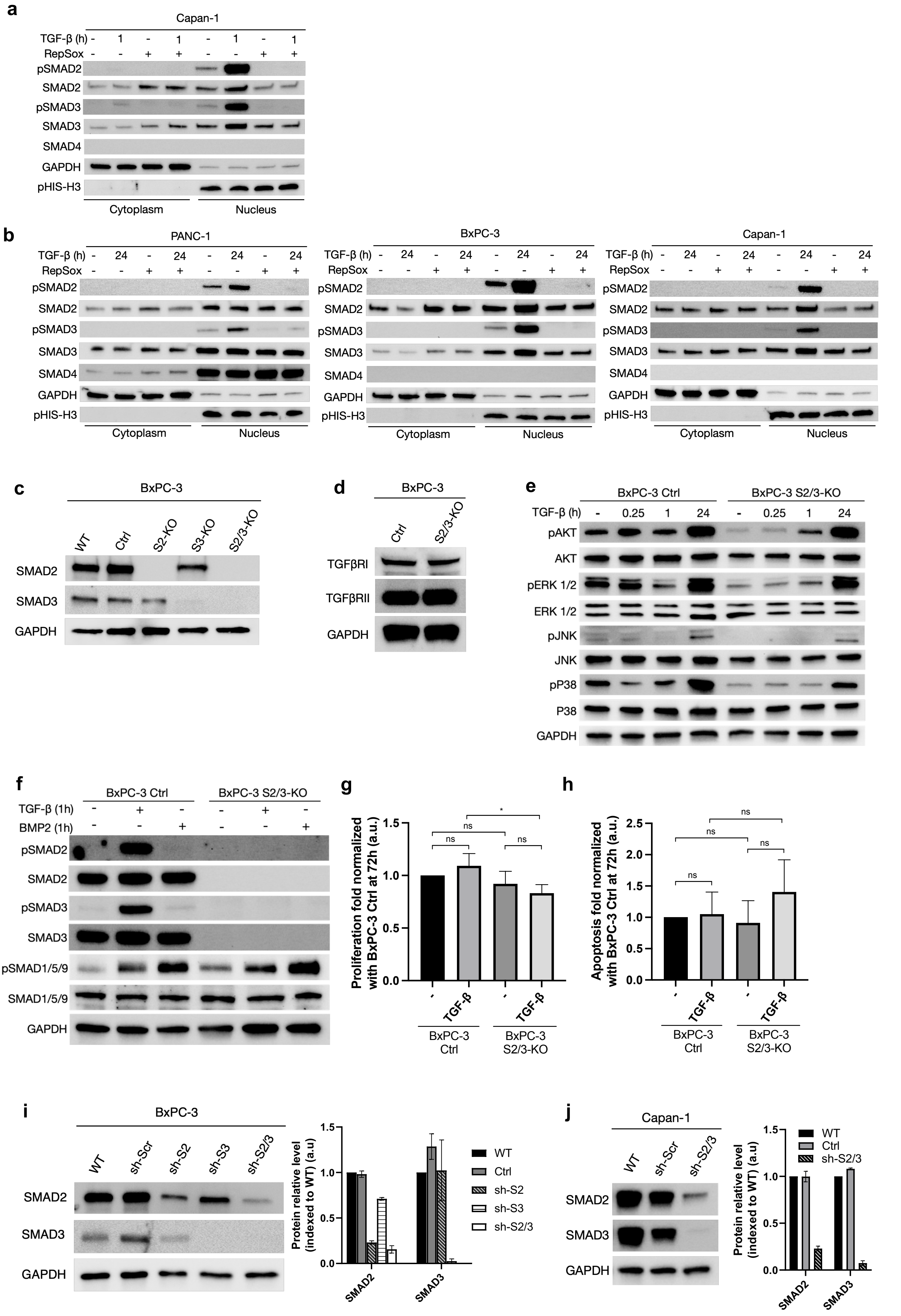
a
Capan-1
TGF-β (h)
RepSox
pSMAD2
SMAD2
pSMAD3
SMAD3
SMAD4
GAPDH
pHIS-H3
Cytoplasm
Nucleus
b
PANC-1
BxPC-3
Capan-1
c
BxPC-3
WT
Ctrl
S2-KO
S3-KO
S2/3-KO
d
TGFβRI
TGFβRII
e
BxPC-3 Ctrl
BxPC-3 S2/3-KO
pAKT
AKT
pERK 1/2
ERK 1/2
pJNK
JNK
pP38
P38
f
TGF-β (1h)
BMP2 (1h)
pSMAD1/5/9
SMAD1/5/9
g
Proliferation fold normalized with BxPC-3 Ctrl at 72h (a.u.)
ns
*
h
Apoptosis fold normalized with BxPC-3 Ctrl at 72h (a.u.)
i
sh-Scr
sh-S2
sh-S3
sh-S2/3
Protein relative level (indexed to WT) (a.u)
j
Capan-1

Figure S1 – Generation of a double SMAD2/SMAD3 knockout/knockdown in BxPC-3 and Capan-1 (SMAD4^{-}negative) pancreatic cancer cell lines. a. Immunoblot of phospho-SMAD2 (pSMAD2), SMAD2, phospho-SMAD3 (pSMAD3), SMAD3 and SMAD4 after treatment with TGF-β and/or TGF-βR1 kinase activity inhibitor (RepSox) for 1 h on cytoplasmic (GAPDH as control) and nuclear fractions (phospho-histone H3 (pHIS-H3) as control) prepared from SMAD4-negative Capan-1 cells. One representative image out of three is shown. **b.** Immunoblot of phospho-SMAD2 (pSMAD2), SMAD2, phospho-SMAD3 (pSMAD3), SMAD3 and SMAD4 after treatment with TGF-β and/or TGFβR1 kinase activity inhibitor (RepSox) for 24 h on cytoplasmic (GAPDH as control) and nuclear fractions (phospho-histone H3 (pHIS-H3) as control) prepared from SMAD4-positive PANC-1 cells and SMAD4-negative BxPC-3 and Capan-1 cells. One representative image out of three is shown. **c.** Immunoblot of SMAD2, SMAD3 and GAPDH on WT, control, S2-KO, S3-KO and S2/3-KO BxPC-3 cells, genetically engineered by CRISPR-Cas9. One representative image out of three is shown. **d.** Immunoblot of TGF-β receptors **I** (TGF-βRI) and **II** (TGF-βRII) and GAPDH on control and S2/3-KO BxPC-3 cells (one representative blot out of 3 independent repeats). **e.** Immunoblot of pAKT, AKT, pERK1/2, ERK1/2, pJNK, JNK, pP38, p38 and GAPDH on control and S2/3-KO BxPC-3 cells treated with TGF-β for 15 min, 1 h or 24 h. One representative image out of three is shown. **f.** Immunoblot of pSMAD2, SMAD2, pSMAD3, SMAD3, pSMAD1/5/9 and SMAD1/5/9 and GAPDH on control and S2/3-KO BxPC-3 cells treated or not with TGF-β or BMP2 for 1 h. One representative image out of three is shown. **g.** Proliferation analysis of control and S2/3-KO BxPC-3 cells treated or not with TGF-β for 72 h. Means +/- SEM are shown (n=3). One-tailed Wilcoxon/Mann-Whitney test; *, p-value < 0.05; ns, not significant. **h.** Apoptosis rate analysis of BxPC-3 control and S2/3-KO BxPC-3 cells treated or not with TGF-β for 72 h. Means +/- SEM are shown (n=3). One-tailed Wilcoxon/Mann-Whitney test; ns, not significant. **i.** Immunoblot of SMAD2, SMAD3 and GAPDH on WT, control (sh-Scr), S2-KD (sh-S2), S3-KD (sh-S3) and S2/3-KD (sh-S2/3) BxPC-3 cells, transfected with specific shRNA targeting SMAD2 or SMAD3. One representative image out of three is shown. SMAD2 and SMAD3 were quantified from three independent experiments and represented as graphs of mean values +/- SD. **j.** Immunoblot of SMAD2, SMAD3 and GAPDH on WT, control (sh-Scr), and S2/3-KD (sh-S2/3) Capan-1 cells. One representative image out of three is shown. SMAD2 and SMAD3 were quantified from three independent experiments and represented as graphs of mean values +/- SD.

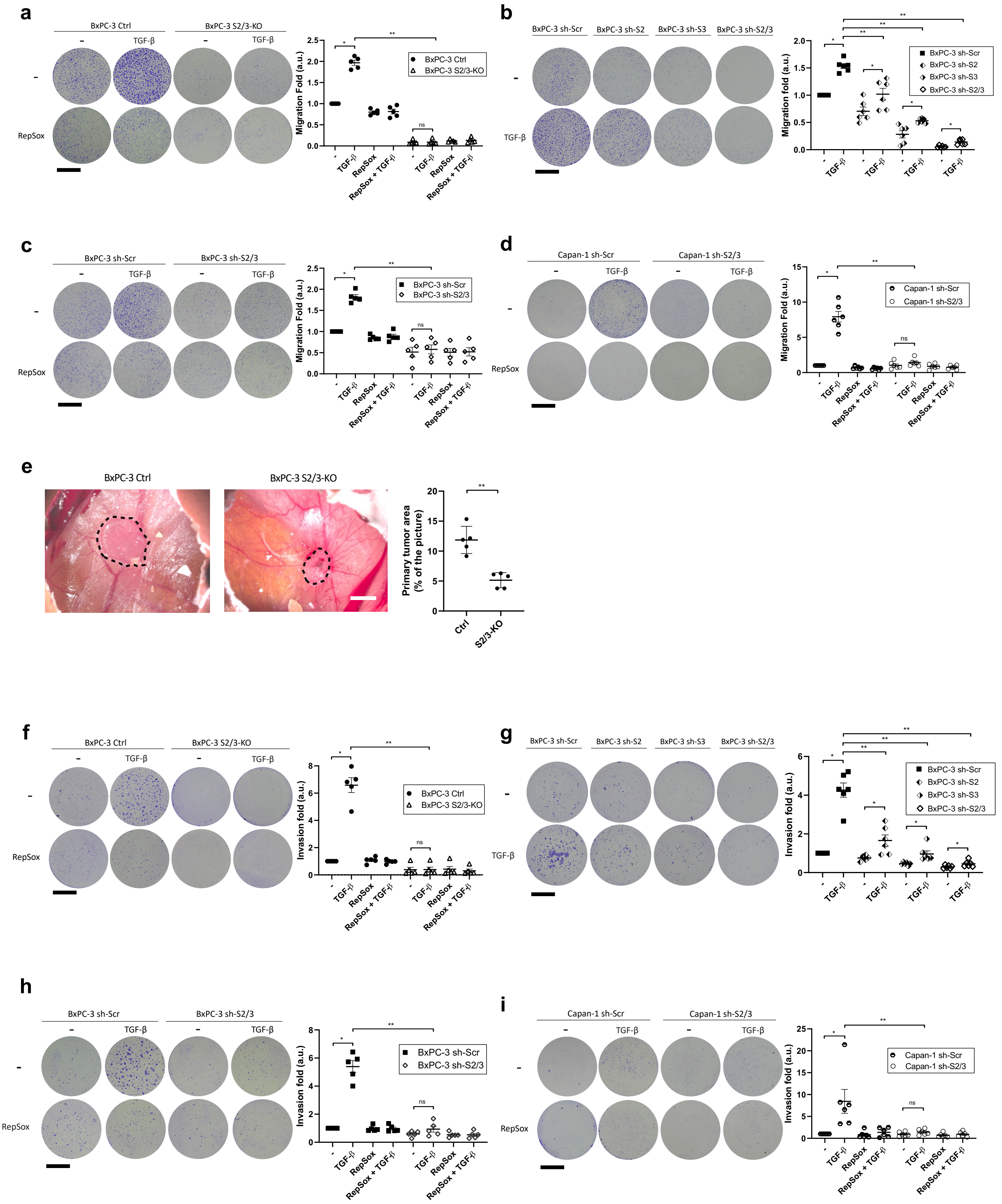
a
BxPC-3 Ctrl
BxPC-3 S2/3-KO
-
TGF-β
RepSox
Migration Fold (a.u.)
BxPC-3 Ctrl
BxPC-3 S2/3-KO
RepSox + TGF-β
ns
b
BxPC-3 sh-Scr
BxPC-3 sh-S2
BxPC-3 sh-S3
BxPC-3 sh-S2/3
Migration fold (a.u.)
c
BxPC-3 sh-Scr
BxPC-3 sh-S2/3
Migration Fold (a.u.)
d
Capan-1 sh-Scr
Capan-1 sh-S2/3
Migration Fold (a.u.)
e
BxPC-3 Ctrl
BxPC-3 S2/3-KO
Primary tumor area (% of the picture)
Ctrl
S2/3-KO
f
BxPC-3 Ctrl
BxPC-3 S2/3-KO
Invasion fold (a.u.)
g
BxPC-3 sh-Scr
BxPC-3 sh-S2
BxPC-3 sh-S3
BxPC-3 sh-S2/3
Invasion fold (a.u.)
h
BxPC-3 sh-Scr
BxPC-3 sh-S2/3
Invasion fold (a.u.)
i
Capan-1 sh-Scr
Capan-1 sh-S2/3
Invasion fold (a.u.)

Figure S2 – SMAD2 and SMAD3 are crucial in the migratory and invasive potential of BxPC-3 and Capan-1 (SMAD4-negative) cells in response to TGF-β. a. Images of a transwell migration assay with control and S2/3-KO BxPC-3 cells cultured for 24 h in the presence or absence of TGF-β and/or RepSox, the TGF-βR1 kinase activity inhibitor. Scale bar = 3mm. Means +/- SEM are shown (n=5). One-tailed Wilcoxon/Mann-Whitney test; **, p-value <0.01; *, p-value <0.05; *ns*, not significant. **b.** Images of a transwell migration assay with control (sh-Scr), S2-KD (sh-S2), S3-KD (sh-S3) and S2/3-KD (sh-S2/3) BxPC-3 cells cultured for 24 h in the presence or absence of TGF-β. Scale bar = 3mm. Means +/- SEM are shown (n=6). One-tailed Wilcoxon/Mann-Whitney test; **, p-value <0.01; *, p-value <0.05. **c.** Images of a transwell migration assay with control (sh-Scr) and S2/3-KD (sh-S2/3) BxPC-3 cells cultured for 24 h in the presence or absence of TGF-β and/or RepSox, the TGF-βR1 kinase activity inhibitor. Scale bar = 3mm. Means +/- SEM are shown (n=5). One-tailed Wilcoxon/Mann-Whitney test; **, p-value <0.01; *, p-value <0.05; *ns*, not significant. **d.** Images of a transwell migration assay with control (sh-Scr) and S2/3-KD (sh-S2/3) Capan-1 cells cultured for 72 h in the presence or absence of TGF-β and/or RepSox, the TGF-βR1 kinase activity inhibitor. Scale bar = 3mm. Means +/- SEM are shown (n=6). One-tailed Wilcoxon/Mann-Whitney test; **, p-value <0.01; *, p-value <0.05; *ns*, not significant. **e.** Images of control or S2/3-KO tumors, 7 days after engraftment on chicken embryo chorioallantoic membranes. Scale bar = 5mm. Tumor mean area (horizontal bar) +/- SD is represented. One-tailed Wilcoxon/Mann-Whitney; **, p-value < 0.01. **f.** Images of transwell invasion assay in matrigel with control and S2/3-KO BxPC-3 cells cultured for 72 h in the presence or absence of TGF-β and/or RepSox. Scale bar = 3mm. Graphs represent mean values (horizontal bar) +/- SEM (n=5). One-tailed Wilcoxon/Mann-Whitney test; **, p-value <0.01; *, p-value <0.05; *ns*, not significant. **g.** Images of a transwell invasion assay with control (sh-Scr), S2-KD (sh-S2), S3-KD (sh-S3) and S2/3-KD (sh-S2/3) BxPC-3 cells cultured for 72 h in the presence or absence of TGF-β. Scale bar = 3mm. Graphs represent mean values (horizontal bar) +/- SEM (n=6). One-tailed Wilcoxon/Mann-Whitney test; **, p-value <0.01; *, p-value <0.05. **h.** Images of a transwell invasion assay with control (sh-Scr) and S2/3-KD (sh-S2/3) BxPC-3 cells cultured for 72 h in the presence or absence of TGF-β and/or RepSox, the TGF-βR1 kinase activity inhibitor. Scale bar = 3mm. Graphs represent mean values (horizontal bar) +/- SEM (n=5). One-tailed Wilcoxon/Mann-Whitney test; **, p-value <0.01; *, p-value <0,05; *ns*, not significant. **i.** Images of a transwell invasion assay with control (sh-Scr) and S2/3-KD (sh-S2/3) Capan-1 cells cultured for 96 h in the presence or absence of TGF-β and/or RepSox, the TGF-βR1 kinase activity inhibitor. Scale bar = 3mm. Graphs represent mean values (horizontal bar) +/- SEM (n=6). One-tailed Wilcoxon/Mann-Whitney test; **, p-value <0.01; *, p-value <0,05; *ns*, not significant.Figure S2

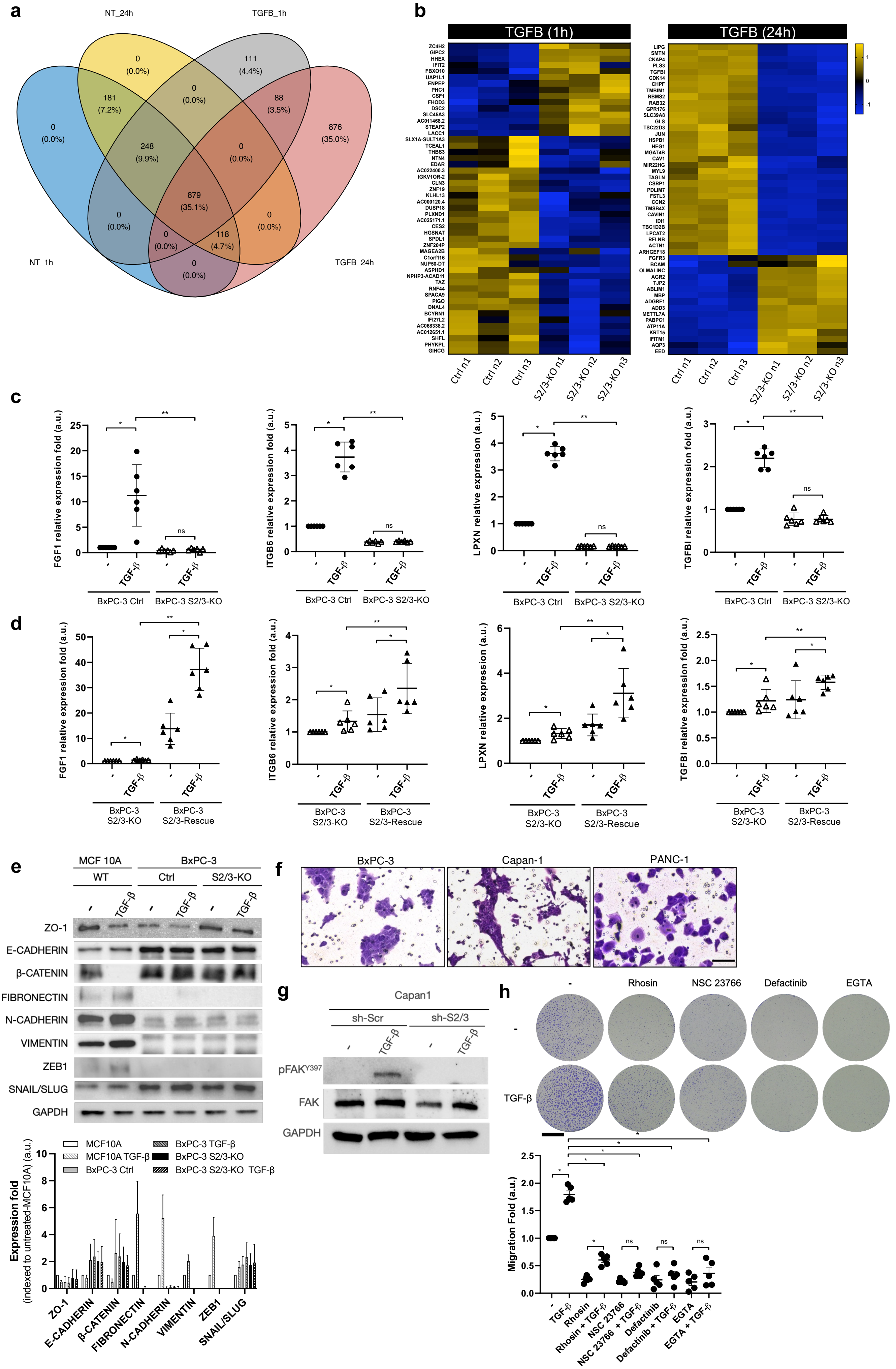
a
NT_24h
TGFB_1h
NT_1h
TGFB_24h
0 (0.0%)
111 (4.4%)
181 (7.2%)
0 (0.0%)
88 (3.5%)
0 (0.0%)
876 (35.0%)
248 (9.9%)
0 (0.0%)
879 (35.1%)
0 (0.0%)
0 (0.0%)
0 (0.0%)
118 (4.7%)
0 (0.0%)
b
TGFB (1h)
TGFB (24h)
Ctrl n1
Ctrl n2
Ctrl n3
S2/3-KO n1
S2/3-KO n2
S2/3-KO n3
c
FGF1 relative expression fold (a.u.)
ITGB6 relative expression fold (a.u.)
LPXN relative expression (a.u.)
TGFBI relative expression fold (a.u.)
TGF-β
BxPC-3 Ctrl
BxPC-3 S2/3-KO
ns
d
BxPC-3 S2/3-KO
BxPC-3 S2/3-Rescue
e
MCF 10A
BxPC-3
WT
Ctrl
S2/3-KO
ZO-1
E-CADHERIN
β-CATENIN
FIBRONECTIN
N-CADHERIN
VIMENTIN
ZEB1
SNAIL/SLUG
GAPDH
MCF10A
MCF10A TGF-β
BxPC-3 Ctrl
BxPC-3 TGF-β
BxPC-3 S2/3-KO
BxPC-3 S2/3-KO TGF-β
Expression fold (indexed to untreated-MCF10A) (a.u.)
f
BxPC-3
Capan-1
PANC-1
g
Capan1
sh-Scr
sh-S2/3
pFAKY397
FAK
GAPDH
h
Rhosin
NSC 23766
Defactinib
EGTA
TGF-β
Migration Fold (a.u.)
Rhosin + TGF-β
NSC 23766 + TGF-β
Defactinib + TGF-β
EGTA + TGF-β

Figure S3 – SMAD2/3 initiate a collective migration transcriptional program in response of TGF-β in a SMAD4-null PDAC context. a. Venn diagram showing the intersection of genes with significant differential expression levels (absolute log2 fold change > 1 and adjusted p-value < 0.05) between conditions (control *vs* S2/3-KO) in four sets of experimental design: BxPC-3 cells untreated (after 1 h or 24 h) or treated with TGF-β (after 1 h or 24 h). **b.** Heatmaps showing the top 50 differentially expressed genes, ranked according to their adjusted p-value, between control and S2/3-KO BxPC-3 cells after 1 h or 24 h of TGF-β treatment. Clustering method: Complete; distance: Euclidean. **c.** RT-qPCR of FGF1, ITGB6, LPXN and TGFBI gene expression in control and S2/3-KO BxPC-3 cells, treated or not with TGF- β for 24 h. Graphs represent mean values (horizontal bar) +/- SEM (n=6). One-tailed Wilcoxon/Mann-Whitney test; **, p-value <0.01; *, p-value <0.05; *ns*, not significant. **d.** RT-qPCR of FGF1, ITGB6, LPXN and TGFBI genes expression in S2/3-KO and S2/3-Rescue cells, treated or not with TGF- β for 24 h. Graphs represent mean values (horizontal bar) +/- SEM (n=6). One-tailed Wilcoxon/Mann-Whitney test; **, p-value <0.01; *, p-value <0.05. **e.** Immunoblot of Zonula-occludens-1 (ZO-1), E-CADHERIN, β-CATENIN, FIBRONECTIN, N-CADHERIN, VIMENTIN, ZEB1, SNAIL/SLUG, and GAPDH on MCF 10A ($SMAD4^{+}$ breast cancer cell line), control and S2/3-KO BxPC-3 ($SMAD4^{-}$) cell lines treated or not with TGF-β for 5 days. Quantification was done on three independant experiment and results are presented as mean index +/- SEM to untreated-MCF10A cells. **f.** High magnification of transwell migration assay after TGF-β treatment (24h), showing collective migration of BxPC-3 and Capan-1 but not PANC-1 cells. Black scale bar = 50*µ*m. **g.** Immunoblot of phospho-Focal Adhesion Kinase ($pFAK^{Y397}$), Focal Adhesion Kinase (FAK) and GAPDH on control (sh-Scr) and S2/3-KD (sh-S2/3) Capan-1 cells treated or not with TGF-β for 24 hours. Immunoblot was repeated three times, one representative image is shown. **h.** Images of a transwell migration assay with control BxPC-3 cells cultured for 24 h in the presence or absence of RhoA inhibitor (Rhosin), Rac-1 inhibitor (NSC 23766), phospho-FAK inhibitor (Defactinib), the calcium-chelator (EGTA) and TGF-β. Scale bar = 3mm. Graphs represent mean values +/- SEM (n=5). One-tailed Wilcoxon/Mann-Whitney test; *, p-value <0.05; *ns*, not significant.

a

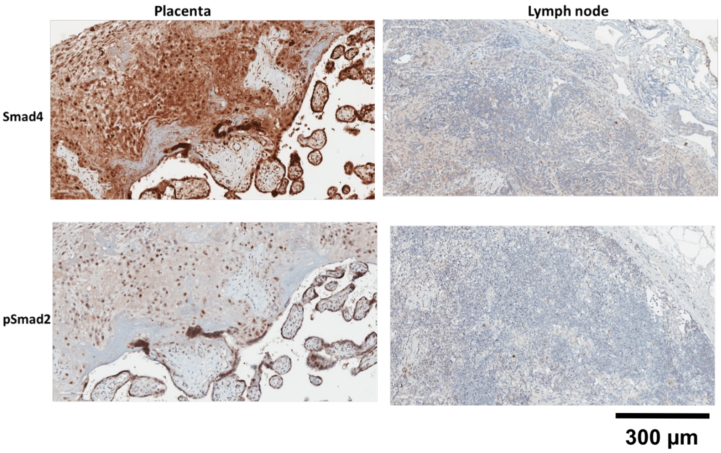

b

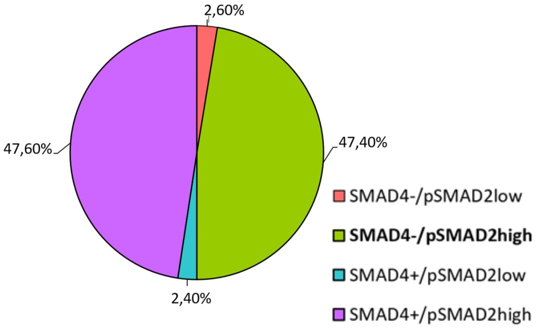

c

	N	Number of events	Median time [95% CI]
SMAD4-/ pSMAD2 low	13	8	3.5 [2.0, NA]
SMAD4-/ pSMAD2 high	240	170	1.7 [1.5, 2.2]
SMAD4+/ pSMAD2 low	12	7	2.2 [2.1, NA]
SMAD4+/ pSMAD2 high	241	158	2.4 [1.9, 2.7]

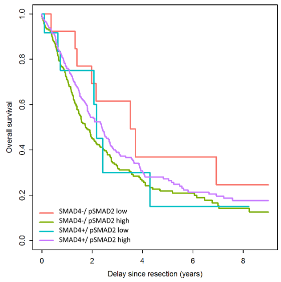

	N	Number of events	Median time [95% CI]
SMAD4-	253	178	1.9 [1.6, 2.3]
SMAD4+	253	165	2.4 [1.9, 2.7]

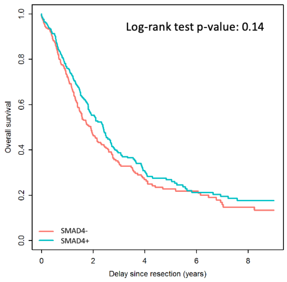

	N	Number of events	Median time [95% CI]
Others	266	173	2.4 [2.0, 2.7]
SMAD4-/ pSMAD2 high	240	170	1.7 [1.5, 2.2]

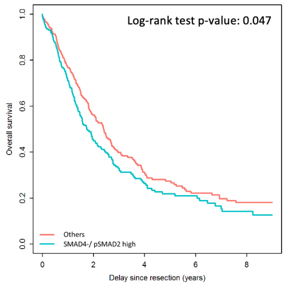

	N	Number of events	Median time [95% CI]
pSMAD2 low	25	15	2.4 [2.1, NA]
pSMAD2 high	481	328	2.0 [1.8, 2.4]

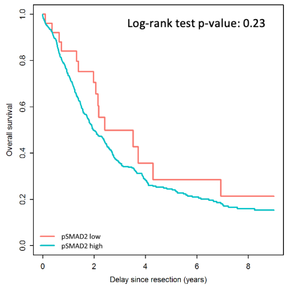

d

Multivariate survival analysis

Variable	HR	95% CI	p-value
Expression pattern (SMAD4/pSMAD2*high* / others)	1.201	[0.966 ; 1.493]	0.099
Sex (male/female)	1.206	[0.971 ; 1.498]	0.091
pTNM II/I Stade	1.397	[1.035 ; 1.885]	**0.029**
pTNM III/IV Stade	2.057	[1.504 ; 2.813]	**<0.001**
Resection (R0/R1)	1.187	[0.952 ; 1.479]	0.128
Perineural Invasion (yes/no)	1.355	[0.976 ; 1.881]	0.069
Necrosis (yes/no)	1.783	[1.436 ; 2.216]	**<0.001**

e

Group	No Smad4$^-$/pSmad2^{high} tumors N = 266	Smad4$^-$/pSmad2^{high} tumors N = 240
pTNM Stage (2017)		
I-II	195 (58.4%)	139 (41.6%)
III-IV	71 (41.3%)	101 (58.7%)
T Status (2017)		
1	44 (53.7%)	38 (46.3%)
2	175 (53.8%)	150 (46.2%)
3	45 (47.9%)	49 (52.1%)
4	2 (50.0%)	2 (50.0%)
N Status (2017)		
0	73 (56.2%)	57 (43.8%)
1	127 (59.1%)	88 (40.9%)
2	66 (41.0%)	95 (59.0%)

Figure S4 – Distribution and multivariate analyses of clinical data from 506 PDAC patients

a. Immunohistochemistry for SMAD4 and pSMAD2 in Negative (lymphocytes in the lymph node) and positive (trophoblastic cells of the placenta) control tissues. Representative images are shown.

b. Graphic representation of the percentage of each group of human PDAC according to SMAD4 and pSMAD2 status. **c.** Survival curves of patient cohort considering each group of human PDAC according to SMAD4 and pSMAD2 status or only SMAD4 expression, or only pSMAD2 level, or the SMAD4$^-$/pSMAD2^{high} tumors *versus* the other group. **d.** Multivariate survival analysis including the model of pTNM staging. **e.** Tumor distribution according to the pTNM stage, the T status and the N status are correlated with the SMAD4$^-$/pSMAD2^{high}.

Differentially expressed genes between control and S2/3-KO BxPC3 cells after TGF-β1 treatment.

TGFB_1h (111)	
AC011468.2	AL109976.1
PAGE1	DNAL4
CCR5AS	PLXND1
EBI3	CARF
LINC02015	TENT5B
OXCT2	IFI27L2
AZGP1	RNF44
AL596214.1	LRRC37A3
AC104083.1	ZNF19
AGAP5	THAP7-AS1
BEST1	HNF4G
AC091390.5	SHFL
CXCL2	SPACA9
FBXO2	SRCAP
RAET1K	PHYKPL
STX19	THBS3
ARHGEF37	EDAR
GIPC2	AC008443.1
IL31RA	ZNF204P
ENPEP	MAGEA2B
FBXO10	INPP5J
SLC45A3	TGM1
STEAP2	MAST1
CDYL2	AC068338.2
UAP1L1	AC008267.5
LACC1	SLX1A-SULT1A3
ZC4H2	AC092720.3
PHC1	AL512274.1
HHEX	AC104695.2
DSC2	AC022826.2
CSF1	HLA-F-AS1
MAP6D1	WHAMMP3
FHOD3	FAM229A
MYO16	COL5A3
IL17RE	SCG5
IFIT2	AC000120.4
ASPHD1	LINC02009
PIGQ	SH3BGR
DUSP18	LMF1
ZNF599	AOC1
CES2	GPR1
AC025171.1	MTTP
BCYRN1	AC022400.3
KLHL13	GIHCG
C1orf116	OR2A20P
AL132712.2	ADORA2A
BX284668.5	AC123768.2
LBHD1	AC093010.3
SPDL1	AC074141.1
NTN4	AC114267.1
TMEM44-AS1	AC012651.1
HGSNAT	AC079594.2
CLN3	AL137060.6
TCEAL1	IGKV1OR-2
TAZ	NPHP3-ACAD11
NUP50-DT	

TGFB_1hTGFB_24h (88)	
ZNF816-ZNF321P	NUAK1
MGAM2	KCNJ15
COLEC11	F3
CH25H	AMIGO2
MUC5B	ZSWIM4
CLPSL1	ROBO1
FAM184B	TMEM92
CPZ	PPP1R14B-AS1
LAMB4	MSX2
N4BP2L1	CAMK2N1
LINC00342	LHX5
SLC16A6	STXBP1
AC016588.2	CAMK2N2
AC090517.5	SLC4A3
AC006504.5	KIAA1191
EIF4E3	RPL26L1
ZSCAN12P1	TRIM7
CALCRL	BHLHE40
DYNC2H1	FAM174B
H2BC4	EN1
CDH6	SIK1B
MIR600HG	CRADD
DEPTOR	PPP1R3F
PEG10	TRIM52-AS1
FSIP2	RIPK3
OBSCN	RTN4R
TCN2	GJB4
RTKN2	RHOB
ZFPM2-AS1	ANKDD1A
ANKRD46	PMEPA1
GRHL1	AL162258.1
ZSCAN31	LINC02084
MCPH1	LINC02551
NFKBIZ	LINC02762
C20orf197	BMERB1
AL109918.1	AC140479.2
DDN	IGFL1
PFKP	PCDH18
TMEM231	AQP10
ZNF688	KRBA2
FAF2	MYO1F
NMB	C5AR2
ATP2C2	MSC-AS1
SFN	APCDD1

TGFB_24h (876)							
RCSD1	COBL	TMPRSS5	ZNF165	PHLDA1	FLRT3	CXXC5	SULT1A3
NAALAD2	CYP3A5	GPR160	PPARGC1B	GCSHP5	MAP1LC3B	GOLGA6L5P	LGALS7B
AC020915.5	AL590708.1	SCEL	FGD3	PXDC1	GDPD1	SLC31A2	CHRNB4
HS6ST2	UNC5B-AS1	HCP5	PRKG2	RIPOR1	MIR193BHG	ZNF710-AS1	ROS1
CABP4	AC100860.1	TMEM255A	AC008014.1	GABARAPL1	ELK3	DISP2	DIO2
AC110619.1	C9orf152	FGFR3	LINC01089	RARS1	IFFO2	HIC1	LPXN
AC162151.2	LYZ	PPM1H	PABPC1	TRABD2A	UBLCP1	FAM166C	GJB2
ELF5	KLRC2	B4GALNT3	ADIRF	EPPK1	MEX3B	COL1A1	ZNF365
FAM96AP2	METTL7A	EVI2B	MZF1	C1orf122	CRIP2	TSPAN5	FGF1
DMD	SNAI3	CCDC80	ILDR1	TRBC2	EML6	KPNA7	CFAP57
AC136475.3	CCL22	MAP3K5	MNS1	FHL2	SLC22A4	CYP26B1	KIAA1755
BCAT1	RALGPS1	TXK	ABLIM1	TMEM98	KLHL25	STAT4	NDP
RAB34	RN7SL2	ARHGAP30	AKR1C3	ADCY7	RNF130	GLIPR2	AC114501.2
AL138885.4	SOX21-AS1	RAB3A	LRTOMT	SLC36A1	MYADM	SLC1A4	NDRG4
SLC6A20	LINC02635	SASH1	SYDE2	ZFP1	ITPR2	ESAM	FAM20A
LINC02377	GDF6	H2AC19	CXADR	PLAAT1	SLC5A12	MIR22HG	PDLIM3
AC016682.1	MMRN2	PHEX	SYTL5	CLTB	LRRC8C	ACTN1	TFEB
STRA6	CYP4B1	TMEM63C	RAD9A	RAB11FIP3	AP000769.1	AC090994.1	NID1
CCL17	ERP27	CLCA4	CEMIP	TCF7L1	ZNF561-AS1	MFSD2A	AC012645.1
SLC26A11	KCNJ2-AS1	LINC01186	GAS6-AS1	TMBIM1	SNAI2	RABGAP1L-DT	HTR1D
FMO3	KRT15	MALAT1	DDX12P	DNAJB5	SIPA1	FSTL3	CRYBA2
LINC02257	AC027020.2	ITGA9-AS1	PER2	GNG2	STARD9	FILIP1L	MAF
PRR15L	MCPH1-AS1	PTPRZ1	NUTM2D	KLF7	AC008687.7	CCDC74A	PLPP4
OFCC1	AC188616.1	KCNH3	CTSC	AP3B2	LAMC2	LPAR5	IL2RB
AC097709.1	EPHA7	HOOK1	DNAAF3	PRDM8	NDRG1	PIK3IP1	CREB5
LINC02863	MYH7B	POF1B	ADD3	INTS12	SMTN	MFAP3L	NKX6-1
BX470111.1	CAPN8	GPD1L	LLGL2	GLA	LIPG	PRSS27	AQP1
AC021733.4	AC140479.7	EXOC6	ANK1	RBMS2	RAB32	SHISA4	MYCT1
BMP5	CA9	PRODH	AC005083.1	VGLL3	NPHP1	LACTB	CSDC2
FAM183A	C15orf62	NEAT1	CHEK2	TPM4	CCDC92	PLEKHG1	NOG
F5	OLMALINC	AC008771.1	AC009005.1	RAB24	EMP3	SOX9	TAGLN
AC016877.3	BCAS1	ARRB2	RDH13	PDPN	TNFAIP1	PTGS2	TMSB4XP6
FUT9	PLAC8	SLC25A21	RCCD1	FAM177A1	STAT5A	FBXO39	AC117378.1
FMO6P	LCN2	AP001273.1	LRRC61	UBE2D3	ANTXR2	MIR181A2HG	NKD1
LYNX1-SLURP2	TGFB3	C11orf54	EZR	FBLN5	MSTO2P	SERPINE2	DYSF
CCL2	AC068896.3	AC108860.2	PCBP1-AS1	BCL11B	P4HA2	AC139795.2	MYL7
SLC5A5	ALDH1A1	PLD6	IKZF2	PLK3	MGLL	AC011462.5	AC037198.1
AC092811.1	STEAP4	KRT13	PLEKHH1	TPCN1	RRAS	FES	AL590004.3
LINC00615	FGFBP3	AC007342.5	HDAC9	CSRP1	SLC46A3	TNFRSF19	AC027682.6
LINC00574	ACSS1	SPIRE2	H2BC11	TIMP1	AL391422.4	LINC00313	AC093724.3
AOAH	PRICKLE4	PARD6B	AC015660.1	KLF6	DMBT1	HOTAIR	MMP24
PLEKHS1	AC002066.1	AL603750.1	NOTCH3	DUSP8	MICAL1	PROC	PSTPIP1
B3GALT5	AC006504.7	IFITM1	AFDN	TTC7B	FRMD6	POU6F1	DKK2
SV2A	MUC1	KLLN	AC093525.6	SLC9A1	HSPB1	LINC00862	ODF3L1
AC025154.2	AL355512.1	ARHGEF16	SPTLC3	CAVIN3	VSIG1	PDLIM7	SIRPB1
CD93	CROCC	CYP4F26P	TFPI2	TMX4	CLIC3	AKAP12	SLC8A2
VSIG2	HPGD	KIAA1324	GKAP1	ST3GAL5	TSC22D3	LPCAT2	ADAM19
TMC1	DEFB1	ALDH3B1	GSDMC	MEX3A	RTN2	ADAMTS6	AC018978.1
TMEM169	AP002478.1	GVQW3	IQANK1	TMEM45A	AL354953.1	FAM66B	NPAS3
BPIFB1	AC138150.2	AL080276.2	WDR31	AC023043.1	RNF122	HOXC8	SIAH3
VIL1	TNNC1	CPT1A	CD82	CASZ1	MCC	RRAD	PCDHGB1
SNX31	AL157829.1	E2F8	CPVL	DGKA	PPP2R5B	ITGA5	AC007744.1
HOXA5	TENT5C	H1-2	IL4R	CCM2	CORO1A	SRRM3	CHST11
USHBP1	FAAH2	CNTNAP3B	TUBB2A	CALD1	RGS3	FOXL1	EFNA2
FAM90A1	SECTM1	ICA1L	CCPG1	PRPS1	ARHGAP24	GBP1	C2orf88
FAM95B1	SLC47A2	ELL3	FGD6	CDC42EP1	B4GALT6	AL356056.2	PODNL1
LINC02747	FMO5	HOXA1	TRNP1	SAMD9L	KLC3	FKBP1B	INSL4
MMEL1	SOX2-OT	CEP126	MT1X	PWWP2A	PTPRK	SPOCD1	FBLN2
FFAR4	IGSF10	LRATD1	EFNB1	ZFPM1	APBB2	MIR100HG	ADAMTS7
AL133227.1	AL596202.1	SLC25A45	NHP2	CAV1	SLC39A8	ABLIM3	HMCN1
TFF3	MAST4-AS1	THEM6	C11orf45	RNASE7	EDIL3	TGM2	FOLR3
RIMKLA	TPRG1	C15orf41	SYT7	STOML1	JUN	PPP1R36	AC008105.3
CACNA2D4	FA2H	HPDL	CHST14	NLRP1	FKBP14	SLC41A2	HTN1
AL078587.2	AC044860.1	MBP	PINK1	CLK4	CHPF	SEMA3C	FGF5
KRT4	RASSF5	KCNJ2	LAMTOR3	DNASE1L1	CAVIN1	ASGR1	AC138894.1
EVPLL	SSBP2	NOTCH2NLA	B3GLCT	CA2	TGFBI	LINC01776	AP000695.1
B3GALT5-AS1	LINC02617	SPINK5	SLC12A4	NIPAL4	MSN	HEG1	DNAH17
REEP2	CLDN16	WNT4	ARL2BP	KCNC3	ULBP1	COL8A2	CST6
MFSD4A	FBXO43	FAM171B	NEDD9	APLP1	ACKR3	LINC00933	AGMO
PIGY	NR6A1	DNAJC3-DT	FAXC	ST20-MTHFS	SLC16A2	CDH2	CALML3
MYO3B	AC083973.1	AP002495.1	DEF8	MLLT11	B4GALNT1	PMP22	RHOJ
SCN9A	RN7SL1	BCAM	AC026877.1	SLC26A2	DQX1	POU3F1	CGB8
AC006460.1	APOBEC3B	STC1	IDI1	PLEK2	GPR176	RFLNB	BEAN1
ATP6V1B1	AC103718.1	IL20RA	ATG4A	SEMA4F	CCDC71L	MYL9	THSD8
FOXJ1	AC007342.8	ASB9	TSPAN33	OSTC	SNAI3-AS1	CGB7	ANK2
SOD3	ELFN1-AS1	TJP2	NRGN	CDON	LARGE1	PDE2A	PCDHGC5
ADH7	LINC01232	LIPH	PTPRE	HOXC13	PTPRB	AC002456.1	AC008687.6
CBFA2T3	C21orf59-TCP10L	DERL3	ZNF319	GLS	MAMLD1	C1QL1	C11orf44
IL12A	H4-16	GRB7	FBLIM1	WASHC4	ALDH1L2	LHB	CSPG4P11
FP565260.3	AC020551.1	RHPN2	MAGEA2	RNF145	TFPI	TRPC1	SIRPAP1
MAB21L4	CASTOR3	PIBF1	KLF12	GPRIN1	PTPN21	BTBD19	AC020907.1
CELP	H2BC18	GULP1	CU633904.2	TEAD2	CD274	KIF26B	AC119044.1
AC104024.3	RAB19	STRBP	GADD45A	FAM214B	FHL3	GPR132	MSC
AC090023.2	AC010655.4	GLS2	MEAK7	RNF217-AS1	FAM241A	PADI3	TRHDE
NAALADL2	TMEM139	AC007342.9	LRP12	MGAT4B	ST6GAL1	CMTM3	DDX11L1
AC111149.2	UPK2	BMP8B	APLF	SYNPO	ATP10D	TUBA1A	GOLGA8G
LINC01559	ALDH1A2	PRR15	VKORC1	ADSS1	JAK3	NKILA	LINC01934
CYP1A1	AL035661.1	ZDHHC23	VEGFC	ST20-AS1	TRAM1	AL451123.1	CD30A
UBR5-AS1	LINC01977	PKIB	CDKN1A	C12orf75	SPHK1	LINC01137	AC016582.1
H19	LYPD6B	KIAA1958	SERINC2	ARHGEF18	CDK14	AC074143.1	RGS18
LINC00589	LY6E-DT	DMC1	STX11	GALNT1	C5AR1	CCDC74B	CDK5R2
AC011294.1	GARNL3	AC108047.1	FURIN	FAT1	SNPH	AP000695.2	AC010894.3
TNNT3	ANKRD33B	LMO7	TMSB4X	STOM	MIR1915HG	TMEM121	AL031681.3
ARNT2	AC007342.4	ZPLD1	CEP112	C2orf27A	TBC1D2B	HOXC13-AS	HOXC5
ZBTB32	AC096677.1	WNK2	WIPI1	BPGM	AC022613.1	STOX2	STK32A
AL118508.2	BCL6	TCN1	APH1B	PPP1R1C	LINC02381	SH2D3C	DAZL
FLG	SPRR1B	LUZP2	MBOAT2	OLR1	HYI	PCOTH	AC010343.3
FAT3	RPS10P7	EED	PML	KCTD17	BEND7	ABLIM2	AL109809.4
CYP4X1	SPAG17	SLC16A12	FAR2	PCDHB2	MATN3	GFPT2	CU639417.3
AC004870.2	TCTE3	RNA5-8SN2	CYLD	KIF21B	GPR157	ACP5	GOLGA8F
LINC01958	KLF15	SMPDL3A	LCAT	CYP4F11	PLS3	AMTN	TNFAIP6
GCNT3	TMEM52	IL1RN	ZYX	CKAP4	MOCS1	GPR156	AC008687.1
ADGRF1	LINC01948	PRAG1	MOB3B	IL1B	HAPLN3	PDCD1LG2	FCRLA
LINC00242	AC012368.1	ATP11A	FERMT2	TMOD2	FKBP7	CSPG4	ADORA1
COL1A2	H2BU1	FLOT2	IL13RA1	GPR137B	AC008687.4	MYH16	HHIP
COLEC10	TMEM170B	HS6ST1	POU2F2	UBTD2	AC093673.1	FAM131C	WSCD2
SLCO4C1	CLTRN	FLVCR2	ADA	CCDC88B	CARMIL2	CCN2	
AC138123.1	AC068580.2	SAPCD2	ITGAV	B3GNT9	AL590822.3	KCNH4	
AGR2	AQP3	SLC25A25-AS1	ERFE	SYDE1	ADGRB2	ITGB6	
CCL20	LINC00261	AP001931.2	LOXL2	TLR6	THSD1	VSNL1	

Table S1. Differentially expressed genes between control and S2/3-KO BxPc-3 cells after TGF-β1 treatement

SMAD2/3 putative targets in response to TGF-β1, in the absence of SMAD4.

SMAD2/3 targets after TGF-β1 treatment, in the absence of SMAD4 (144)		
AC020915.5	FILIP1L	NLRP1
ABLIM2	FLG	NOG
AC004870.2	FN1	ODF3L1
AC008687.4	FOXL1	PCDHGC5
AC010343.3	GCNT3	PDLIM7
AC011294.1	GDPD5	PIK3IP1
AC037198.1	GFPT2	PLEKHG1
AC090994.1	GJB2	PODXL
AC114501.2	GOLGA8G	POU2F2
ACKR3	GPR132	PROC
ADAM19	GPR157	PRODH
ADAMTS6	H19	PTGS2
ADH7	HAPLN3	PTPRB
AGR2	HIC1	PTPRE
AKAP12	HOXC13	PTPRK
AKR1B10	HOXC13-AS	RHOV
AL358334.2	IL20RA	RNASE7
AL451123.1	ITGB6	RRAD
AMTN	KCNJ15	SEMA3C
ANKRD33B	KCNJ2	SERPINE1
APCDD1L-DT	KLF7	SERPINE2
AQP1	KLHL25	SH2D3C
ATP6V1B1	KPNA7	SIAH3
C5AR1	KRT13	SLC26A2
C5AR2	KRT15	SLC46A3
CASZ1	LAMC2	SOX9
CCDC74A	LARGE1	SPHK1
CCDC80	LARGE2	SPOCD1
CDKN1A	LGALS7B	STAT4
CGB5	LINC00313	STEAP4
CGB7	LINC00589	SYNPO
CGB8	LINC00862	TAGLN
CHST11	LINC02551	TFEB
CLCA4	LIPG	TGFBI
CLIC3	LNCOG	TGM2
COL1A1	LOXL2	TLR6
CORO1A	LPAR5	TMEM45A
CRIP2	LPCAT2	TMEM92
CSPG4	LPXN	TNFRSF19
CYP26B1	MAF	TUBA1A
DAZL	MATN3	TXK
DEFB1	MICAL1	VGLL1
DISP2	MIR100HG	VGLL3
DYSF	MIR22HG	WNT4
EFNA2	MUC1	
ELFN1	MYADM	
ESAM	MYH16	
FAM214B	MYL9	
FBXO39	NDRG4	
FGF1	NKILA	

Table S2. SMAD2/3 putative targets in response to TGF-β1 in the absence of SMAD4

Gene target (human)	Primer sequence (5'-3')
FGF1	F:TACTCTGAGAAGAAGACACC
	R:GCGCTTTCAAGACTAAAGAG
ITGB6	F:TGATCCTTAAGTTGAGACCAG
	R:CTCCTTTATTGTGTTGAGGTC
LPXN	F:GTTAGATGCCTTATTGGAGG
	R:GGACTTGTGTTATCCTGAATAG
TGFBI	F:GGATTGTAACTGTGAACTGTG
	R:CAATGATCTGCTGGATGTTG
GAPDH	F:CGGAGTCAACGGATTTGGTCGTAT
	R:AGCCTTCTCCATGGTGGTGAAGAC

Table S3. Primers sequences used in RT-qPCR

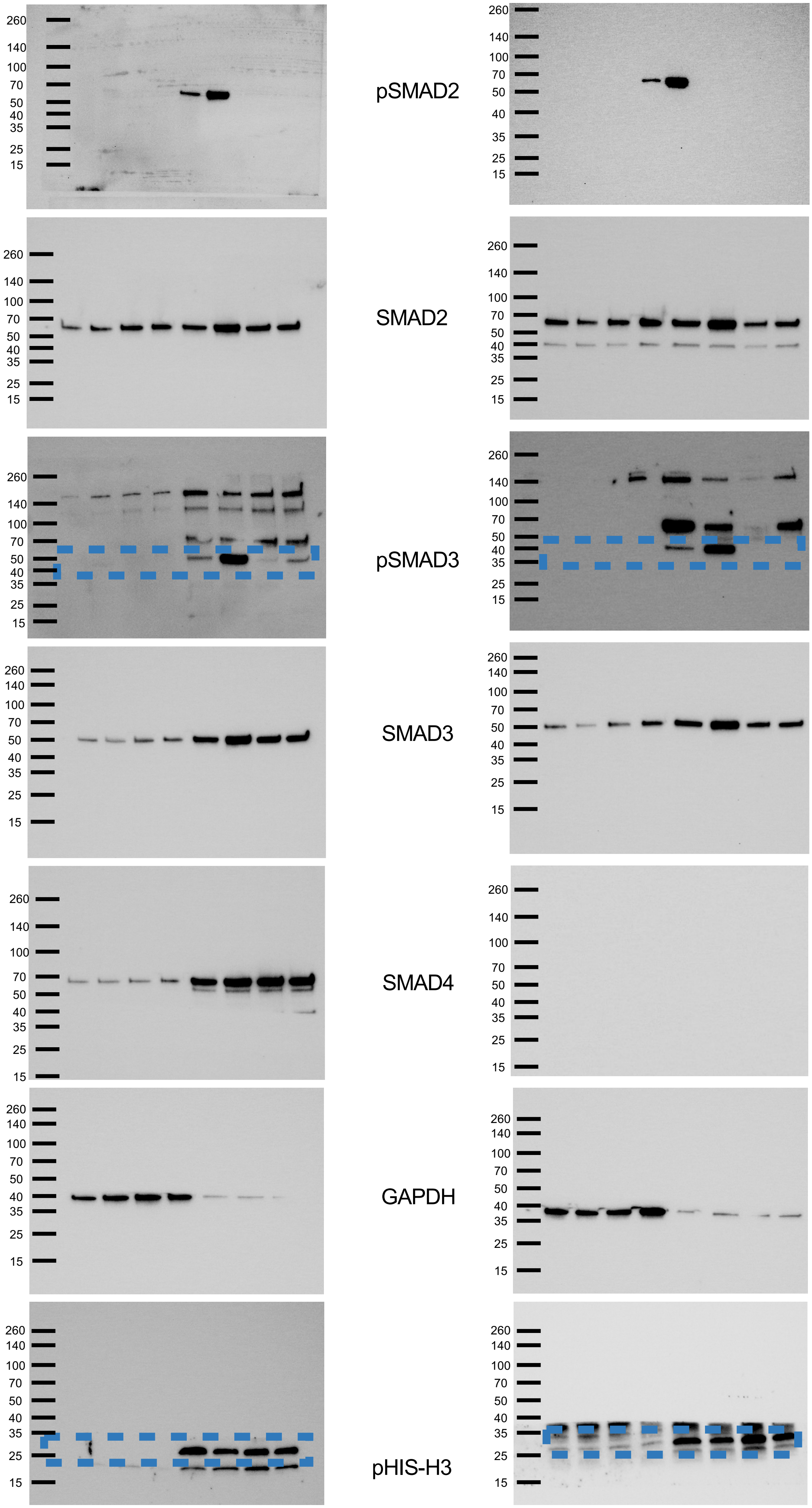
pSMAD2
SMAD2
pSMAD3
SMAD3
SMAD4
GAPDH
pHIS-H3
260
140
100
70
50
40
35
25
15

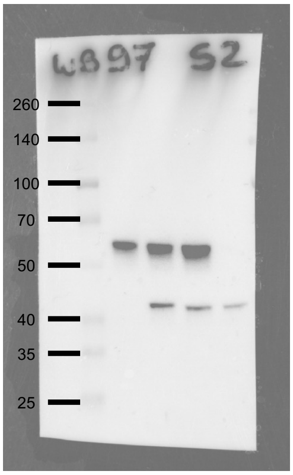

SMAD2

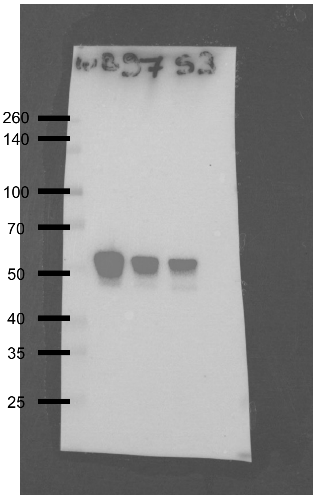

SMAD3

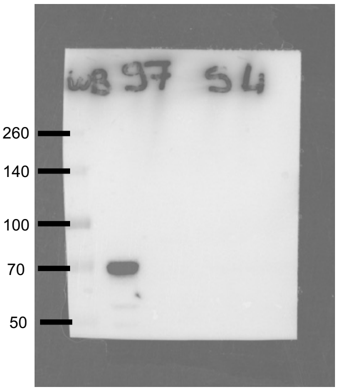

SMAD4

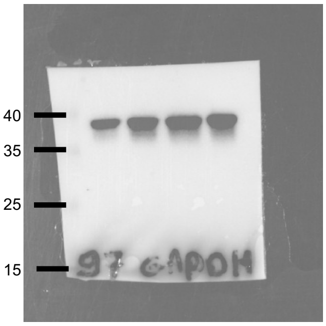

GAPDH

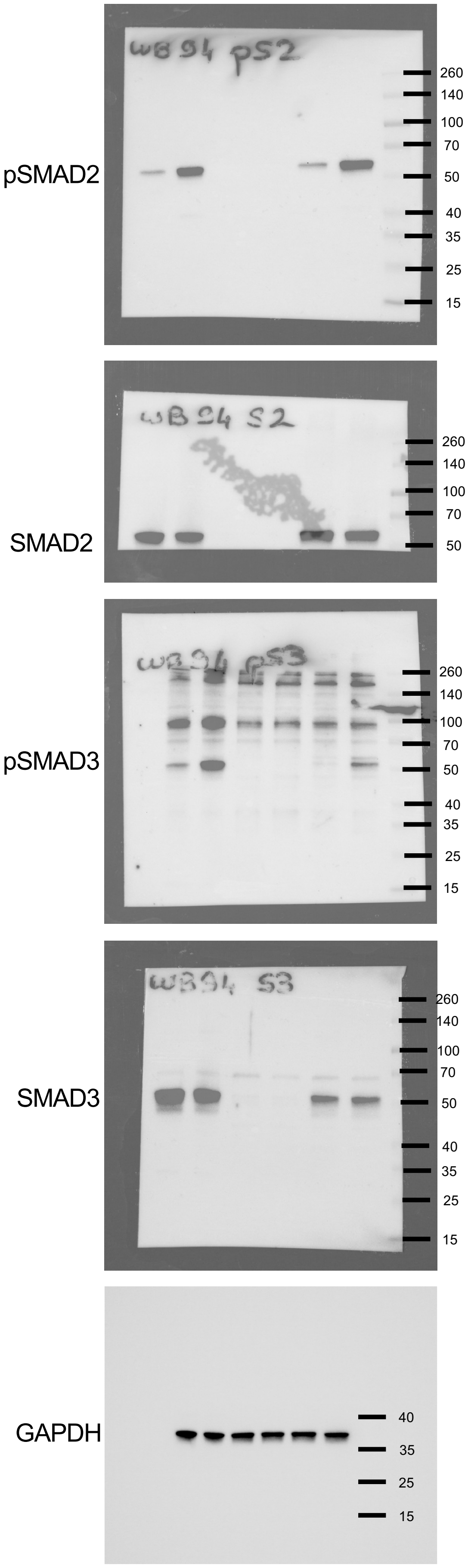
pSMAD2
260
140
100
70
50
40
35
25
15
SMAD2
260
140
100
70
50
pSMAD3
260
140
100
70
50
40
35
25
15
SMAD3
260
140
100
70
50
40
35
25
15
GAPDH
40
35
25
15

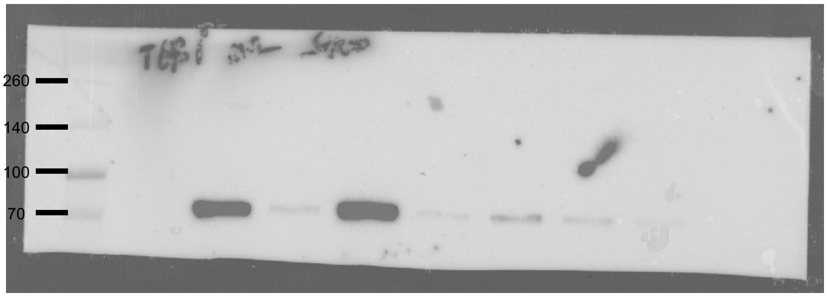

βIG-H3

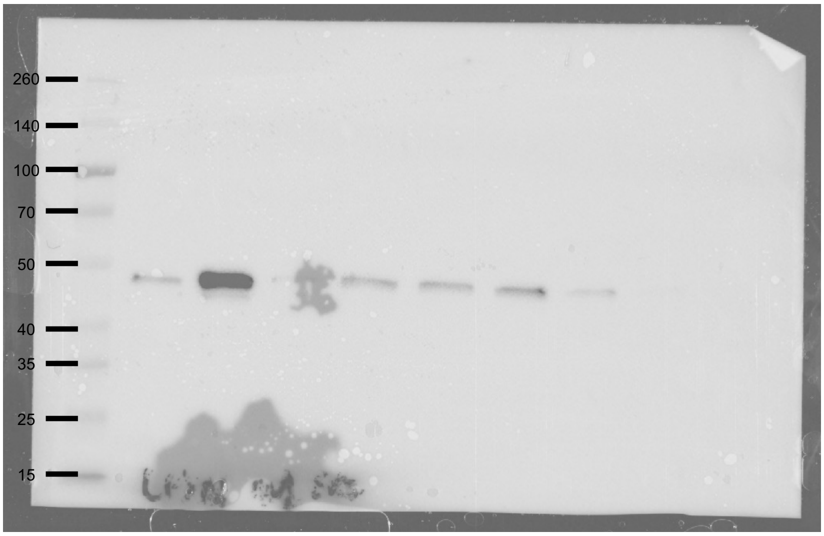

LPXN

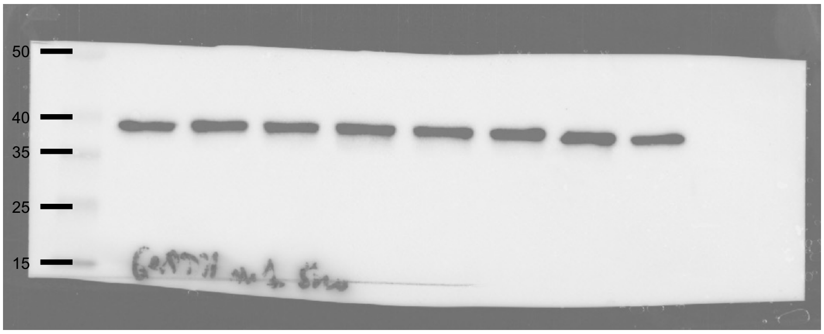

GAPDH

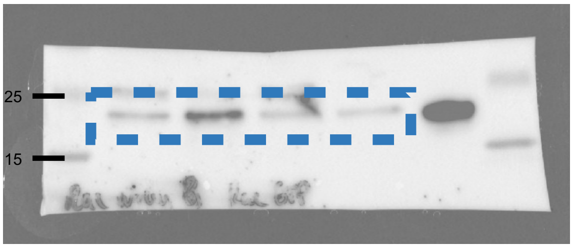

RAC1-GTP

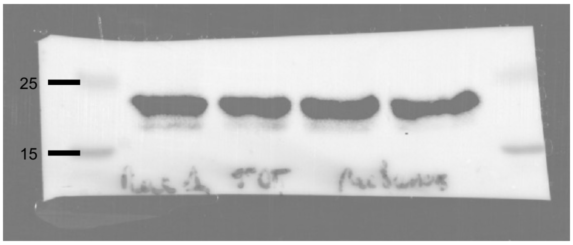

RAC1

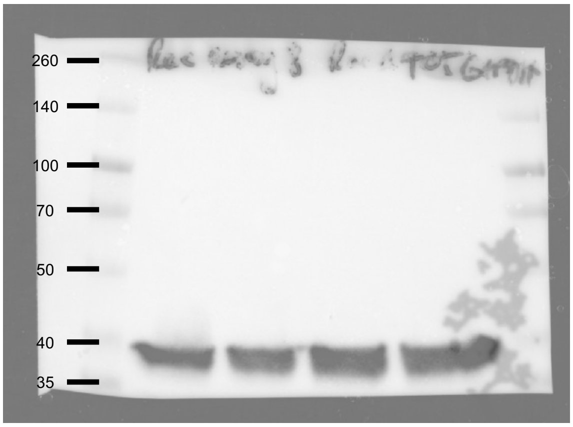

GAPDH

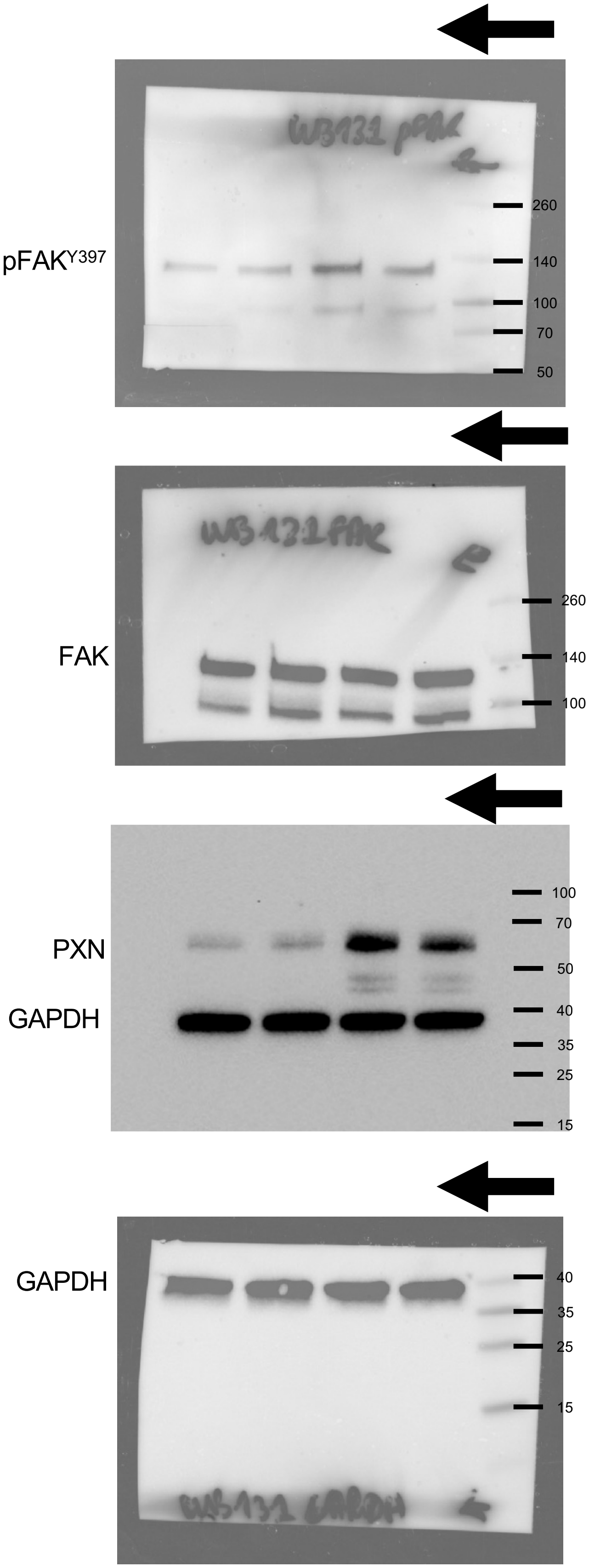

pFAK^{Y397}
260
140
100
70
50
FAK
260
140
100
PXN
GAPDH
100
70
50
40
35
25
15
GAPDH
40
35
25
15

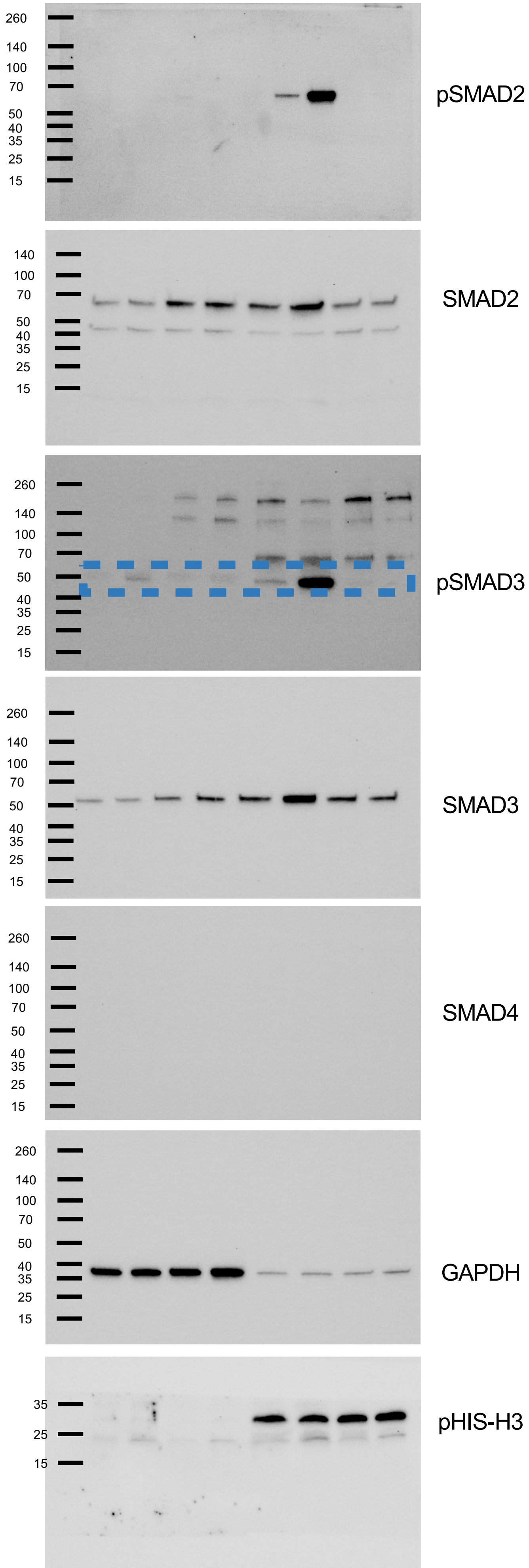
260
140
100
70
50
40
35
25
15
pSMAD2
140
100
70
50
40
35
25
15
SMAD2
260
140
100
70
50
40
35
25
15
pSMAD3
260
140
100
70
50
40
35
25
15
SMAD3
260
140
100
70
50
40
35
25
15
SMAD4
260
140
100
70
50
40
35
25
15
GAPDH
35
25
15
pHIS-H3

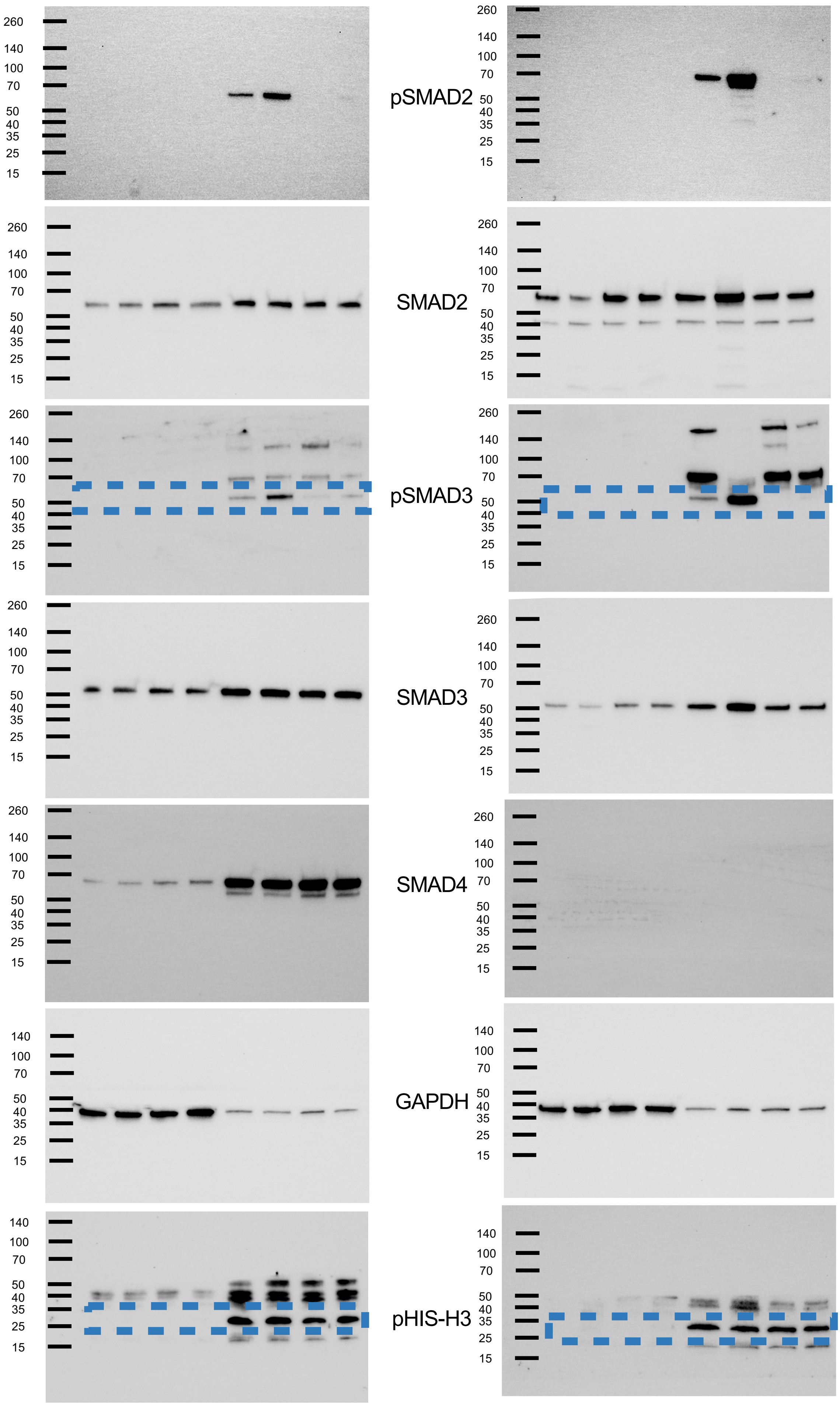

pSMAD2
SMAD2
pSMAD3
SMAD3
SMAD4
GAPDH
pHIS-H3
260
140
100
70
50
40
35
25
15

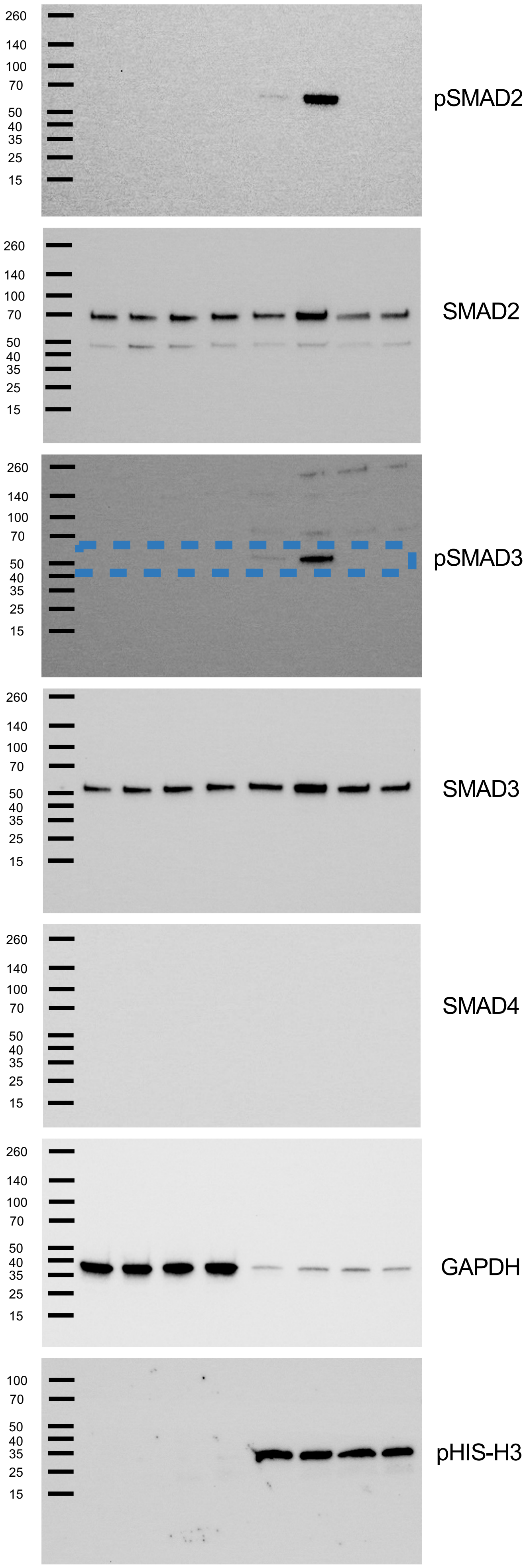

260
140
100
70
50
40
35
25
15
pSMAD2
260
140
100
70
50
40
35
25
15
SMAD2
260
140
100
70
50
40
35
25
15
pSMAD3
260
140
100
70
50
40
35
25
15
SMAD3
260
140
100
70
50
40
35
25
15
SMAD4
260
140
100
70
50
40
35
25
15
GAPDH
100
70
50
40
35
25
15
pHIS-H3

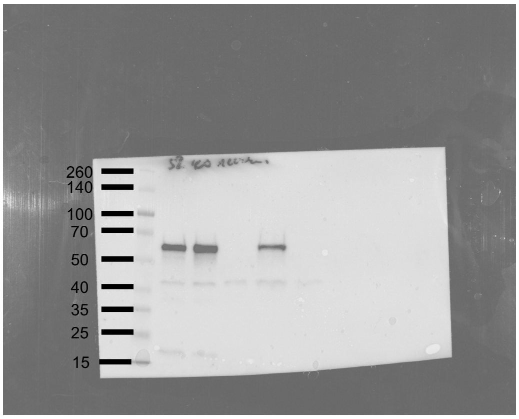

SMAD2

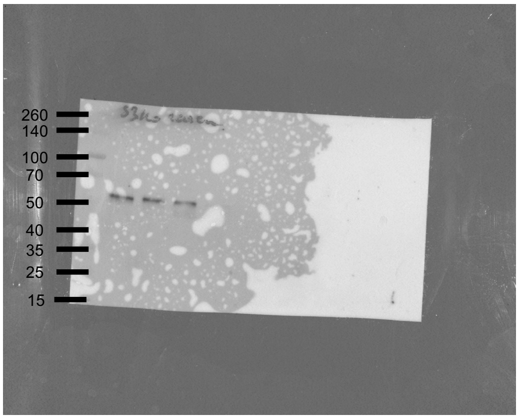

SMAD3

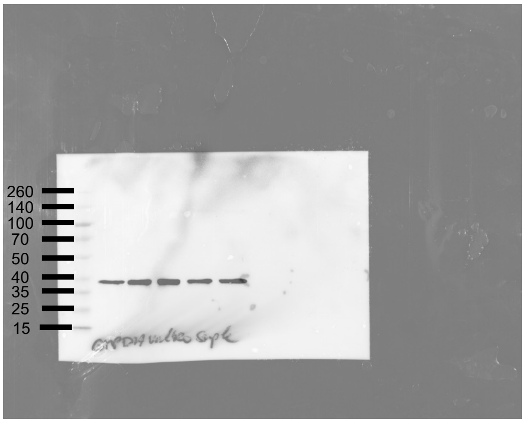

GAPDH

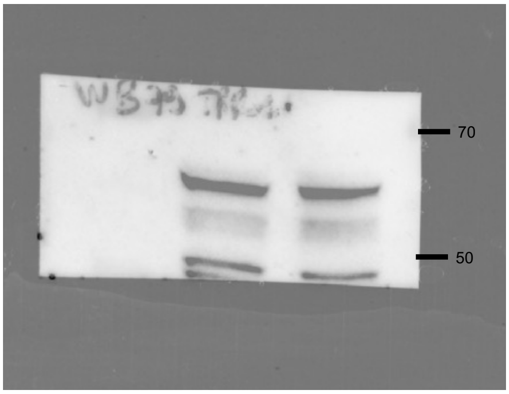

TGFβRI

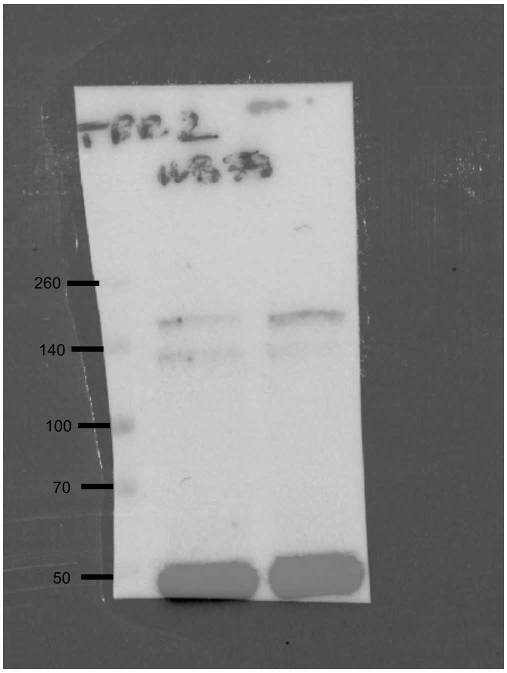

TGFβRII

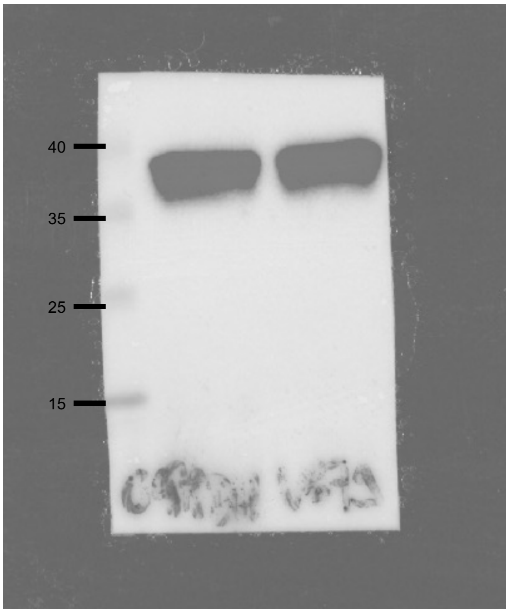

GAPDH

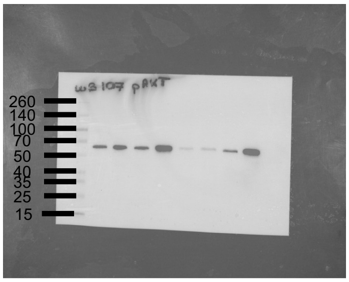

pAKT

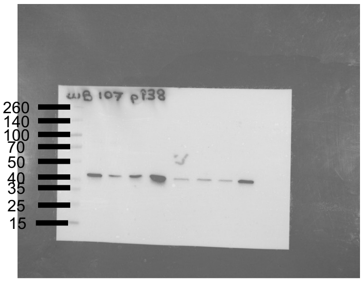

pP38

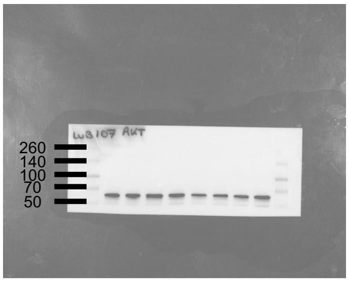

AKT

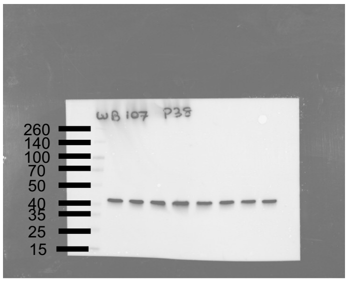

P38

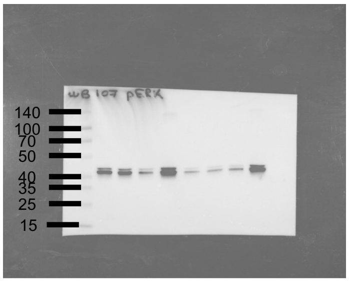

pERK1/2

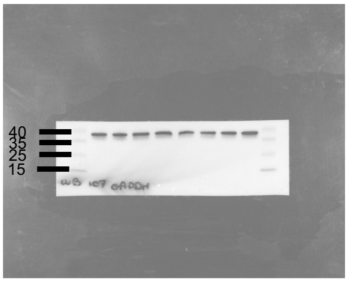

GAPDH

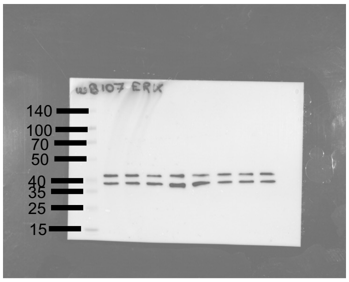

ERK1/2

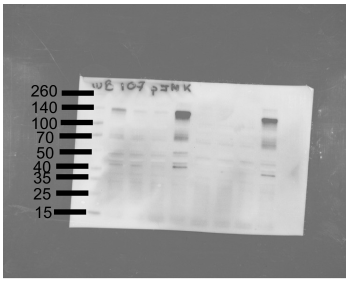

pJNK

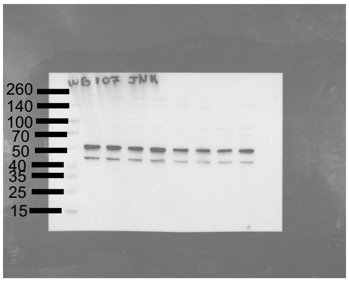

JNK

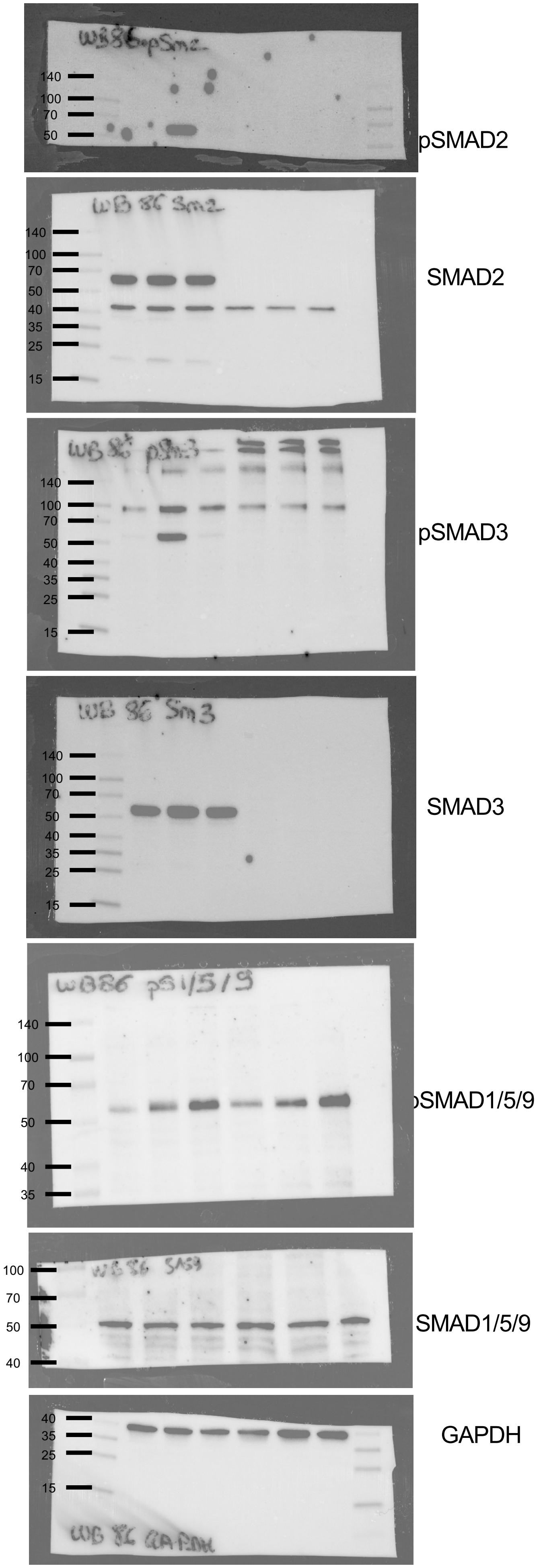
140
100
70
50
pSMAD2
140
100
70
50
40
35
25
15
SMAD2
140
100
70
50
40
35
25
15
pSMAD3
140
100
70
50
40
35
25
15
SMAD3
140
100
70
50
40
35
pSMAD1/5/9
100
70
50
40
SMAD1/5/9
40
35
25
15
GAPDH

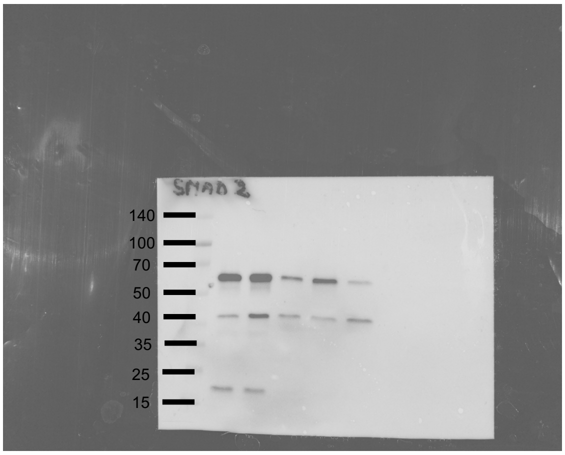

SMAD2

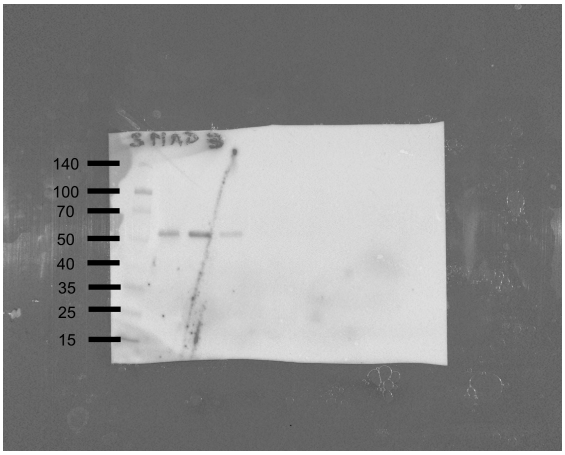

SMAD3

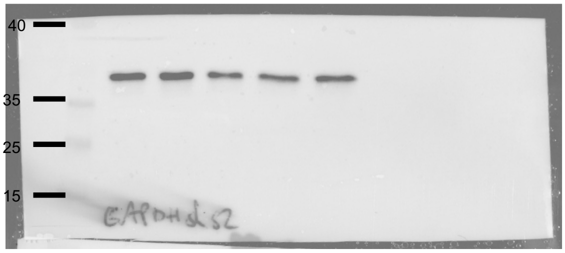

GAPDH

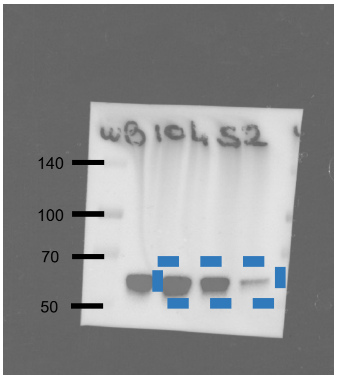

SMAD2

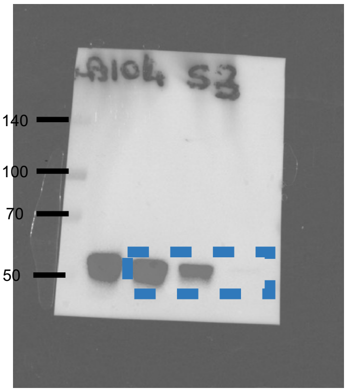

SMAD3

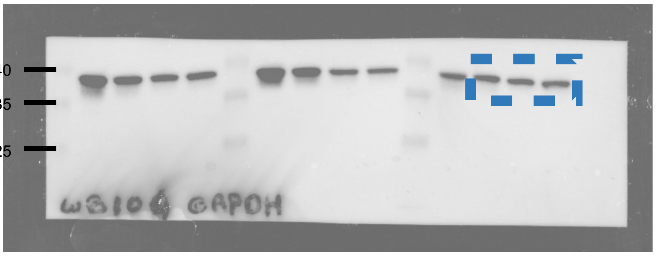

GAPDH

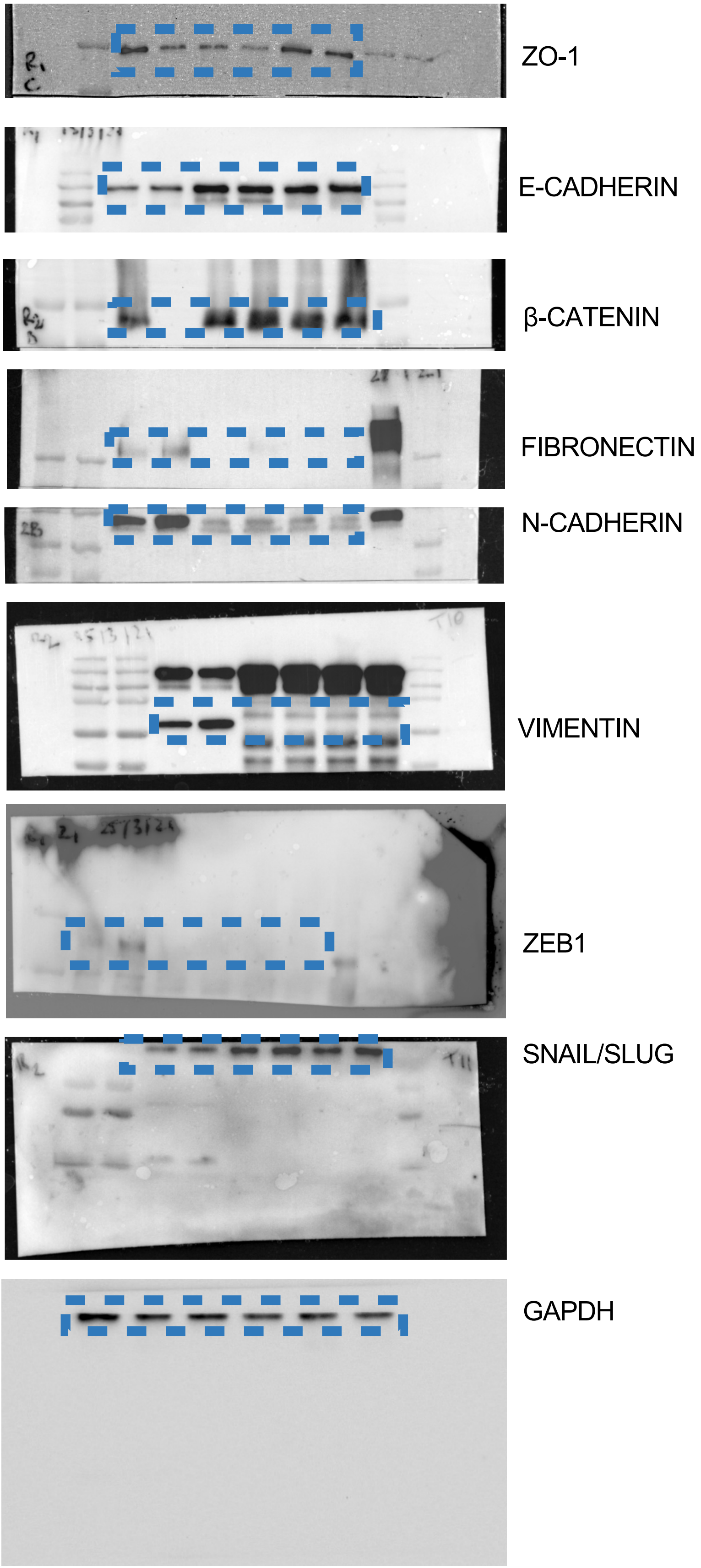
ZO-1
E-CADHERIN
β-CATENIN
FIBRONECTIN
N-CADHERIN
VIMENTIN
ZEB1
SNAIL/SLUG
GAPDH

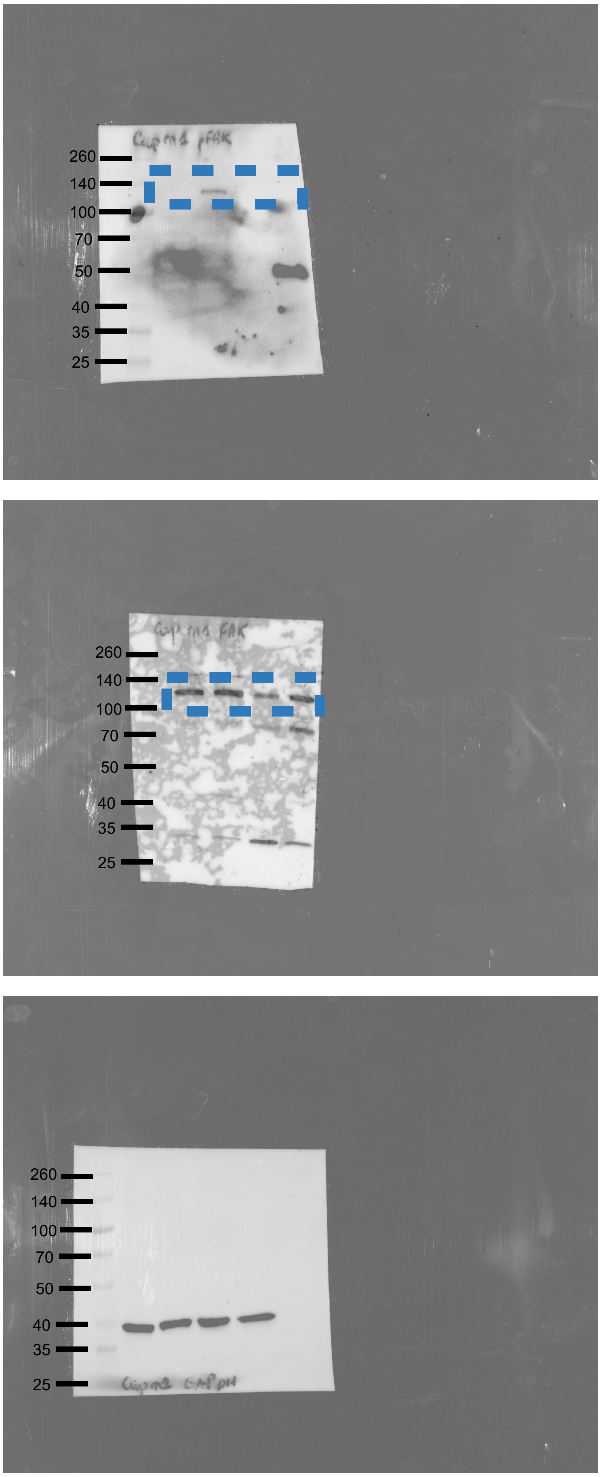

pFAK^{Y397}

FAK

GAPDH

Figure S5 – Unprocessed blots
