# Supplementary material for: SMAD2/3 mediate oncogenic effects of TGF-β in the absence of SMAD4
Source: Commun Biol. 2022 Oct 7;5:1068. doi: 10.1038/s42003-022-03994-6 (PMC9546935; doi:10.1038/s42003-022-03994-6)
Supplement: Supplementary file 2 — Description of Additional Supplementary Files [file 42003_2022_3994_MOESM2_ESM.pdf]

Description of Additional Supplementary Files

File name: Supplementary Data 1
Description: The source data behind the graphs in the paper.

File name: Supplementary Movie 1
Description: BxPC-3 cells showed collective migration phenotype after TGF-β treatment.

File name: Supplementary Movie 2
Description: Capan-1 cells showed collective migration phenotype after TGF-β treatment.
