# Supplementary material for: SMAD2/3 mediate oncogenic effects of TGF-β in the absence of SMAD4
Source: Commun Biol. 2022 Oct 7;5:1068. doi: 10.1038/s42003-022-03994-6 (PMC9546935; doi:10.1038/s42003-022-03994-6)
Supplement: Supplementary file 6 — Reporting Summary [file 42003_2022_3994_MOESM6_ESM.pdf]

nature portfolio

Corresponding author(s): Marie CASTETS

Last updated by author(s): Sep 6, 2022

Reporting Summary

Nature Portfolio wishes to improve the reproducibility of the work that we publish. This form provides structure for consistency and transparency in reporting. For further information on Nature Portfolio policies, see our Editorial Policies and the Editorial Policy Checklist.

Statistics

For all statistical analyses, confirm that the following items are present in the figure legend, table legend, main text, or Methods section.

n/a	Confirmed	
☐	☒	The exact sample size (*n*) for each experimental group/condition, given as a discrete number and unit of measurement
☐	☒	A statement on whether measurements were taken from distinct samples or whether the same sample was measured repeatedly
☐	☒	The statistical test(s) used AND whether they are one- or two-sided<br>*Only common tests should be described solely by name; describe more complex techniques in the Methods section.*
☒	☐	A description of all covariates tested
☐	☒	A description of any assumptions or corrections, such as tests of normality and adjustment for multiple comparisons
☐	☒	A full description of the statistical parameters including central tendency (e.g. means) or other basic estimates (e.g. regression coefficient) AND variation (e.g. standard deviation) or associated estimates of uncertainty (e.g. confidence intervals)
☒	☐	For null hypothesis testing, the test statistic (e.g. *F*, *t*, *r*) with confidence intervals, effect sizes, degrees of freedom and *P* value noted<br>*Give P values as exact values whenever suitable.*
☒	☐	For Bayesian analysis, information on the choice of priors and Markov chain Monte Carlo settings
☒	☐	For hierarchical and complex designs, identification of the appropriate level for tests and full reporting of outcomes
☒	☐	Estimates of effect sizes (e.g. Cohen's *d*, Pearson's *r*), indicating how they were calculated

Our web collection on statistics for biologists contains articles on many of the points above.

Software and code

Policy information about availability of computer code

Data collection	No software used.
Data analysis	For RNA-Seq experiment, gene expression was quantified using Salmon (v1.1.0) (Patro et al, 2017) and the annotation of known genes from Gencode v33 (Frankish et al. 2021). PCA analysis was done with the plotPCA function of the DESeq2 R package (Wickham H, 2016). Unless otherwise specified, the analyses were performed using R (v3.6.1) and illustrations produced with the ggplot 2 (Love et al., 2014) and ggpubr packages. Raw expression data were normalized to their log transcripts per million (logTPM) values before clustering and visualization. For DEG analysis, we used the R package DESeq2 (v1.26). Pathway enrichment analysis was performed with R packages cluster profiler (Gu et al. 2016) (v3.8.1) and org.Hs.eg.db (Yu et al. 2012) (v3.5.0).

For manuscripts utilizing custom algorithms or software that are central to the research but not yet described in published literature, software must be made available to editors and reviewers. We strongly encourage code deposition in a community repository (e.g. GitHub). See the Nature Portfolio guidelines for submitting code & software for further information.

Data

Policy information about availability of data

All manuscripts must include a data availability statement. This statement should provide the following information, where applicable:

- Accession codes, unique identifiers, or web links for publicly available datasets
- A description of any restrictions on data availability
- For clinical datasets or third party data, please ensure that the statement adheres to our policy

The raw date supporting the findings of this study have been deposited in GEO with the accession code GSE178714. These data are currently private until June 2023 but are available from the corresponding author upon request. Whole Genome Sequencing are also available under request.

Human research participants

Policy information about studies involving human research participants and Sex and Gender in Research.

Reporting on sex and gender	*Use the terms sex (biological attribute) and gender (shaped by social and cultural circumstances) carefully in order to avoid confusing both terms. Indicate if findings apply to only one sex or gender; describe whether sex and gender were considered in study design whether sex and/or gender was determined based on self-reporting or assigned and methods used. Provide in the source data disaggregated sex and gender data where this information has been collected, and consent has been obtained for sharing of individual-level data; provide overall numbers in this Reporting Summary. Please state if this information has not been collected. Report sex- and gender-based analyses where performed, justify reasons for lack of sex- and gender-based analysis.*
Population characteristics	*Describe the covariate-relevant population characteristics of the human research participants (e.g. age, genotypic information, past and current diagnosis and treatment categories). If you filled out the behavioural & social sciences study design questions and have nothing to add here, write "See above."*
Recruitment	*Describe how participants were recruited. Outline any potential self-selection bias or other biases that may be present and how these are likely to impact results.*
Ethics oversight	*Identify the organization(s) that approved the study protocol.*

Note that full information on the approval of the study protocol must also be provided in the manuscript.

Field-specific reporting

Please select the one below that is the best fit for your research. If you are not sure, read the appropriate sections before making your selection.

☒ Life sciences ☐ Behavioural & social sciences ☐ Ecological, evolutionary & environmental sciences

For a reference copy of the document with all sections, see nature.com/documents/nr-reporting-summary-flat.pdf

Life sciences study design

All studies must disclose on these points even when the disclosure is negative.

Sample size	Sample size were provided in the manuscript. The sample size were determined based on litterature and standard experimental design. We did not perform sample size calculation.
Data exclusions	No data excluded
Replication	Each experiments was repeated at least three times.
Randomization	No randomization required.
Blinding	All experiments were done and analysed blind.

Reporting for specific materials, systems and methods

We require information from authors about some types of materials, experimental systems and methods used in many studies. Here, indicate whether each material, system or method listed is relevant to your study. If you are not sure if a list item applies to your research, read the appropriate section before selecting a response.

Materials & experimental systems

n/a	Involved in the study
☐	☒ Antibodies
☐	☒ Eukaryotic cell lines
☒	☐ Palaeontology and archaeology
☐	☒ Animals and other organisms
☐	☐ Clinical data
☐	☐ Dual use research of concern

Methods

n/a	Involved in the study
☒	☐ ChIP-seq
☐	☒ Flow cytometry
☒	☐ MRI-based neuroimaging

Antibodies

Antibodies used	Antibodies against SMAD2 (#5339), SMAD3 (#9523), phospho-SMAD2 (Ser465/467) (#3108), phospho-SMAD3 (Ser423/425) (#9520),SMAD2/3 (#8685), SMAD4 (#46535), RAC1 (#4651), !IG-H3 (#5601) and Leupaxin (#59309) were purchased from Cell Signaling. Theanti-GAPDH antibody (#8245), anti-phospho histone H3(#14955), anti-Snail/Slug (#85936), SMAD1/5/9 (#80255) were from Abcam.Anti-Paxilin (PXN) (PA5-111334) and anti-pFAKY397 (44-625G) were from Invitrogen. Anti-vinculin (V9131), anti-FAK (#05-537) and<br>anti-ZEB1 (#HPA027524) were from Sigma. ZO-1 (# 610966), E-cadherin (#610405), !-Catenin (#610154), Fibronectin (#610077) andN-Cadherin (#610920) primary antibodies were from BD Biosciences. Antibody against vimentin was from DAKO (#GA63061). HRPcoupled anti-rabbit secondary antibody were from Immuno Reagents (GtxRb-003-DHPRX). HRP-coupled anti-mouse secondaryantibody were from Dako Cytomation (P0260).
Validation	All antibodies have been previously validated and used in similar assays on human cells, except for SMAD4 and pSMAD2 on humansamples, whose validation is explained in the manuscript (Results and Material/Method).

Eukaryotic cell lines

Policy information about cell lines and Sex and Gender in Research

Cell line source(s)	All cell line were obtained from ATCC.
Authentication	None of the cell lines have been authenticated in our lab since three years
Mycoplasma contamination	All cell lines tested negative for Mycoplasma contamination.
Commonly misidentified lines<br>(See ICLAC register)	*Name any commonly misidentified cell lines used in the study and provide a rationale for their use.*

Animals and other research organisms

Policy information about studies involving animals; ARRIVE guidelines recommended for reporting animal research, and Sex and Gender in Research

Laboratory animals	Fertilized chick eggs (Gallus gallus domesticus) were obtained from EARL Morizau on day 1 of embryonic development andincubated in Anipath facility until day 17 of embryonic development<br><br>(Fli:GFP) CASPER Zebrafish (Danio rerio) embryos were obtained from PRECI (IGFL, ENS Lyon) after the fertilization and raised until 3days post-fertilization
Wild animals	N/A
Reporting on sex	N/A
Field-collected samples	*For laboratory work with field-collected samples, describe all relevant parameters such as housing, maintenance, temperature, photoperiod and end-of-experiment protocol OR state that the study did not involve samples collected from the field.*
Ethics oversight	PRECI and Anipath are two local structures dedicated to animal experimentation, both approved by the French Ministry and inaccordance with the ethical rules in force.<br>The study did not involve wild animals.<br>The study did not involve samples collected from the field.<br>- Local ethical comitee of the Centre Léon Bérard (CEEA10 - "ACCeS" Lyon France) approved the chorioallantoic membrane studyprotocol.<br>- No ethical approval was required for zebrafish embryos experiment until 5 days post-fertilization.

Note that full information on the approval of the study protocol must also be provided in the manuscript.

Clinical data

Policy information about clinical studies

All manuscripts should comply with the ICMJE guidelines for publication of clinical research and a completed CONSORT checklist must be included with all submissions.

Clinical trial registration	The human data come from a database that is declared (MR004 certification n°19-088) and has received a favorable opinion from theCommittee for the protection of persons (CPP) of the Hospices Civils de Lyon (n°19-109).
Study protocol	As it is not a clinical trial under the French law, there is no protocole avalaible in a public data bank
Data collection	All the samples from patients who undergo surgery in one of the two expert centers for pancreatic surgery (Edouard Herriot and Croix Rousse Hospital Digestive surgery unit) of the Hospices Civils de Lyon between the 01st of January 2004 and the 31th ofDecember 2017 for pancreatic ductal adenocarcinoma were included in the cohort. The clinical data were retrieved from the sharedcomputerized medical files of each patient (age at diagnosis, gender, date of diagnosis, date of the latest news or date of death,recurrence) from the 1 november 2018 until the 1st of april 2019.
Outcomes	Primary outcome was to assess the link between SMAD4/pSMAD2 expression pattern and overall survival (cf. Material and Methodsfor statistic analysis and details about the SMAD4/pSMAD2 expression pattern definition).

Dual use research of concern

Policy information about dual use research of concern

Hazards

Could the accidental, deliberate or reckless misuse of agents or technologies generated in the work, or the application of information presented in the manuscript, pose a threat to:

No	Yes	
☒	☐	Public health
☒	☐	National security
☒	☐	Crops and/or livestock
☒	☐	Ecosystems
☒	☐	Any other significant area

Experiments of concern

Does the work involve any of these experiments of concern:

No	Yes	
☒	☐	Demonstrate how to render a vaccine ineffective
☒	☐	Confer resistance to therapeutically useful antibiotics or antiviral agents
☒	☐	Enhance the virulence of a pathogen or render a nonpathogen virulent
☒	☐	Increase transmissibility of a pathogen
☒	☐	Alter the host range of a pathogen
☒	☐	Enable evasion of diagnostic/detection modalities
☒	☐	Enable the weaponization of a biological agent or toxin
☒	☐	Any other potentially harmful combination of experiments and agents

Flow Cytometry

Plots

Confirm that:

☐ The axis labels state the marker and fluorochrome used (e.g. CD4-FITC).

☒ The axis scales are clearly visible. Include numbers along axes only for bottom left plot of group (a 'group' is an analysis of identical markers).

☐ All plots are contour plots with outliers or pseudocolor plots.

☒ A numerical value for number of cells or percentage (with statistics) is provided.

Methodology

Sample preparation	BxPC-3 Cells were obtained from ATCC. Cells were detached, washed and resuspended with Flow Cytometry Staining Buffer (eBioscience #00-4222-26). To assess cell viability, cells were stained with LIVE/DEAD Far Red Dead Cell solution (Invitrogen#L10120). Cells were fixed and permeabilized for 30 min with 1X FoxP3 Fixation/Permeabilization Buffer (eBioscience#00-5523-00), washed, resuspended in 1X Permeabilization Buffer (eBioscience #00-8333-56), then blocked with 5%-FBSPermeabilization Buffer for 30 min. Cells were then incubated with anti-SMAD2/3 antibody for 45 min, washed twice with 1XPermeabilization Buffer, incubated with anti-rabbit Alexa Fluor 488-conjugated antibody (Invitrogen 477 A32731) for 30 minand washed twice with 1X Permeabilization Buffer. Samples were resuspended in Flow Cytometry Staining Buffer beforeanalysis with BD Canto II flow cytometer.
Instrument	BD FACSCanto II Flow Cytometry System was used for data collection.
Software	BD FACS Diva Software were used to collect and analysed the flow cytometry data.
Cell population abundance	The purity of the two samples is almost 100% but not 100% due to fixation, permeabilization or staining failures. Dead cell were removed with LIVE/DEAD Far Red Dead Cell solution.
Gating strategy	The gating strategy for boundaries between positive and negative cells is based on negative control and isotype control of primary antobodies.

☐ Tick this box to confirm that a figure exemplifying the gating strategy is provided in the Supplementary Information.
